# Cognitive change 5+ years since the onset of a psychotic disorder: A systematic review and meta-analysis

**DOI:** 10.1017/S0033291725100627

**Published:** 2025-07-16

**Authors:** Joseph Ghanem, Andrew J. Watson, Samantha Aversa, Christy Au-Yeung, Olivier Percie du Sert, Marie Starzer, Melissa A. Weibell, Helene Gjervig Hansen, Katie M. Lavigne, Martin Lepage

**Affiliations:** 1 Douglas Research Centre, Montréal, QC, Canada; 2 Department of Psychology, McGill University, Montréal, QC, Canada; 3 Department of Clinical, Educational and Health Psychology, University College London, London, UK; 4 South London and Maudsley NHS Foundation Trust, London, UK; 5 Department of Psychiatry, McGill University, Montréal, QC, Canada; 6 Copenhagen Research Center for Mental Health – CORE, Mental Health Center Copenhagen, Mental Health Services in the Capital Region, Copenhagen, Denmark; 7 Department of Clinical Medicine, University of Copenhagen, Copenhagen, Denmark; 8 Regional Centre for Clinical Research in Psychosis, Division of Psychiatry, Stavanger University Hospital, Stavanger, Norway; 9 Faculty of Social Sciences, University of Stavanger, Stavanger, Norway

**Keywords:** longitudinal, meta-analysis, neurocognition, psychotic disorders, schizophrenia

## Abstract

Cognitive impairments are a core feature of psychotic disorders, but their long-term trajectory remains contentious. Previous meta-analyses focused on the first 5 years following psychosis onset. Here, we evaluated the change in cognitive impairments in psychotic disorders with a meta-analysis of studies with follow-ups of 5+ years. Following preregistration, databases were searched for relevant articles until July 2024. Two authors screened the reports for studies reporting on the change in cognitive impairments in global cognition, verbal learning and memory, visual learning and memory, working memory, attention, speed of processing, reasoning and problem-solving, and verbal fluency in individuals with psychotic disorders, with a minimum follow-up of 5 years. Three authors extracted data, and the PRISMA guidelines were followed. Random-effects meta-analyses and moderator analyses were conducted. Twenty-four studies comprising 2,633 patients and 1,019 controls were included in the study. Over an average of 8.46 years, cognitive impairments remained stable in all eight measures: global cognition (*g* = 0.09; 95% CI = 0.03–0.20), verbal memory (*g* = 0.05; 95% CI = −0.11, 0.21), visual memory (*g* = −0.16; 95% CI = −0.35, 0.03), working memory (*g* = 0.03; 95% CI = −0.09, 0.14), attention (*g* = 0.22; 95% CI = −0.36, 0.80), speed of processing (*g* = 0.10; 95% CI = −0.14, 0.35), reasoning and problem-solving (*g* = 0.16; 95% CI = −0.03, 0.35), and verbal fluency (*g* = 0.08; 95% CI = −0.03, 0.19). We conclude that cognitive impairments remain stable over time, consistent with the neurodevelopmental view of psychotic disorders.

## Introduction

Cognitive impairment is a core feature of psychotic disorders and has been the subject of considerable scientific interest (Keefe & Harvey, 2012). The relationship between cognitive impairment, functional outcomes, and negative symptoms is well-documented (Au-Yeung et al., 2023; Lepage, Bodnar, & Bowie, 2014), with some even calling for the classification of schizophrenia as a cognitive illness (Kahn & Keefe, 2013).

Although cognitive impairments are unequivocally present – often as large as 1 standard deviation below the mean of healthy controls when examining antipsychotic-naïve patients with a first episode of psychosis (FEP) (Lee et al., 2024) – their long-term trajectory following psychosis onset remains contentious. Previous meta-analytic studies identified modest improvements in cognition following illness onset (Bora & Murray, 2014; Watson et al., 2022), but the improvements were similar to those of healthy controls and are largely accounted for by practice effects (Watson et al., 2022). Importantly, these reviews focused on the first 5 years following illness onset, leaving the longer-term trajectory of cognition, beyond 5 years, unclear. Recently, a meta-analysis included a few studies with longer follow-up periods but had a median follow-up of 2 years (Catalan et al., 2024). Significantly, these reviews were solely focused on FEP to the exclusion of studies examining schizophrenia spectrum disorders (SSDs) more broadly, despite similar trajectories of cognition over time. Indeed, a general pattern of stability has been observed both in FEP and in people with enduring schizophrenia, at least in the short term (Bora & Murray, 2014; Szöke et al., 2008; Watson et al., 2022).

The literature on cognitive trajectories beyond 5 years is inconsistent. For instance, in one 18-year follow-up study of first-admission psychosis patients, cognitive decline was apparent in most cognitive domains, with a similar rate of change over time across psychotic disorders (Fett et al., 2020). Similarly, one 10-year follow-up study observed that cognition in individuals with schizophrenia declined in several cognitive domains, but this decline was not apparent in those with other psychotic disorders (Zanelli et al., 2019). Conversely, other studies with 10-year follow-up periods revealed a stable cognitive performance (Rodríguez-Sánchez et al., 2022; Sánchez-Torres et al., 2013), and so did studies with 15- and 20-year follow-up periods (Albus et al., 2020; Bonner-Jackson, Grossman, Harrow, & Rosen, 2010), irrespective of diagnostic category.

To provide more definitive conclusions on the evolution of cognitive performance in the longer term, we conducted a meta-analysis and synthesized evidence from studies with a minimum follow-up of 5 years to provide an estimate of the change in cognition over time in global cognition and seven neurocognitive domains, including the six neurocognitive domains outlined by the Measurement and Treatment Research to Improve Cognition In Schizophrenia (Green, Kern, & Heaton, 2004). These domains include verbal learning and memory, visual learning and memory, working memory, the speed of processing, reasoning, and problem-solving, as well as attention and vigilance. We further examined verbal fluency, given evidence for its existence as a separate domain (Dickinson, Ramsey, & Gold, 2007; Fett et al., 2011; Watson et al., 2022). In doing so, we build upon previous meta-analyses that focused on FEP and follow-up periods of 1–5 years. Longer follow-ups are important, as they capture changes through illness progression and are less prone to practice effects, given the longer timeframe. Furthermore, sampling from studies with different age groups and diagnostic categories provides a clearer picture of the long-term evolution of cognition. We additionally aimed to identify moderators of change over time to better characterize the change in cognitive impairment and further understand long-term outcomes.

## Methods

### Search strategy and data extraction

The present review followed the Preferred Reporting Items for Systematic Reviews and Meta-Analyses guidelines (Page et al., 2021) (Supplementary eTable 1), and the protocol was preregistered on PROSPERO (CRD42023447589) in July 2023. The search strategy was guided by an expert librarian, and PsycINFO, MEDLINE, and SCOPUS were searched for relevant articles published between 1999 and July 26, 2023 (search strategy in Supplementary eTable 2). Updated searches were conducted until July 2024. Snowballing was performed by one author (CA) by searching reference lists from previous meta-analyses.

Following duplicate removal, records were moved to Covidence and screened by two authors (JG and SA) with a 10% random overlap. Discrepancies were resolved through discussion with each other and with another author (CA). Inclusion criteria for the studies were: (i) reporting data on individuals with SSDs, FEP, and other psychotic disorders, including affective psychoses; (ii) being published in English; (iii) having a minimum follow-up period of 5 years; and (iv) using validated neuropsychological tests and batteries. In addition, both studies, including controls and studies without controls, were included. Exclusion criteria for the studies were: (i) having follow-ups under 5 years; (ii) reporting data on early or childhood-onset psychosis; and (iii) being unpublished papers, conference abstracts, or reviews. Studies needed to report the sample sizes, means, and standard deviations at baseline and follow-up for the cognitive tests used. Authors were contacted for access to the data if unreported in the published studies. Three authors performed data extraction starting on November 9, 2023 (JG, SA, and CA). When studies included overlapping samples and reported similar cognitive data, the one with the longest follow-up period was selected. However, if studies had overlapping samples but one reported more cognitive data (e.g., reported data on more domains), the latter was included to maximize the number of studies included in each domain meta-analysis.

### Study quality assessment

Three authors (JG, SA, and CA) independently assessed study quality and risk of bias with the Mixed Methods Appraisal Tool (MMAT) (Hong et al., 2018) and two authors (JG and SA) independently reviewed all ratings for accuracy and completeness. MMAT ratings for each individual study, as well as notes on study quality, are presented in Supplementary eTable 3.

### Classification of cognitive tests and effect size computation

Cognitive tests employed by the individual studies were classified into their respective domains based on the literature, a recent meta-analysis (Watson et al., 2022), the compendium of neuropsychological tests (Carone, 2007), and co-authors with expertise in neurocognitive testing (KML and AW) (test allocations presented in Supplementary eTable 4). If studies reported several tests for the same domain, one effect size was computed by averaging the effect sizes, similar to Watson et al. (Watson et al., 2022). The effect sizes of all cognitive domains were pooled together to create a global cognitive effect size. Therefore, for each study, an effect size per domain was extracted (or an average was computed, if several tests were reported per domain), and all domain effect sizes were averaged to provide a single global effect size measure in keeping with the approach and recommendations of previous meta-analyses (Van den Noortgate, López-López, Marín-Martínez, & Sánchez-Meca, 2015; Vita et al., 2024; Watson et al., 2022). The global cognition computation was only performed for studies reporting data on two or more domains. If a study only reported IQ, it was used as an estimate for global cognition. If a study reported both IQ and raw scores for a minimum of two domains, the domain effect sizes were used. If a study reported both raw scores for the individual tests of a domain and an index score for that domain, we used the raw scores of the individual tests to calculate an average effect size. This was done for consistency with test allocation. For instance, some studies may have included in an index measure of attention a measure that we classified as the speed of processing (e.g., TMT-A).

### Statistical analysis

Before the within-subject rate of change meta-analysis in patients and controls, we first established the presence of a cognitive impairment at the first time point by running between-group meta-analyses for each domain. In doing so, we computed standardized mean differences (SMDs) with an adjustment for differences in sample sizes (Hedge’s *g*) for each included domain using the “escalc” function from the “metafor” R package (Viechtbauer, 2010).

Subsequently, we calculated within-subject SMDs for both patients and controls with a similar sample size adjustment (Hedge’s *g*). Because the calculation of within-subject SMDs requires knowledge of the correlation coefficient between the first and second time points, we used the conservative estimate of *r* = 0.65 and conducted sensitivity analyses at the lower and upper bounds of the 95% confidence interval (0.58 and 0.70, respectively), consistent with Catalan et al. (2024).

Patients and controls were first compared on each domain over time using the *Q*-test to assess differences between subgroups. The *Q*-test permits the inclusion of all studies with available data for each group, as opposed to traditional between-group meta-analyses (Watson et al., 2022), but may be underpowered (Cuijpers, Griffin, & Furukawa, 2021). Therefore, to assess the robustness of our results, an additional between-groups meta-analysis was conducted to compare the rate of change over time between patients and controls.

All meta-analyses were conducted using random-effects models due to the expected heterogeneity in effect sizes using the “rma” function from the “metafor” package. No adjustment for multiple comparisons was made, similar to previous reviews (Bora & Murray, 2014; Catalan et al., 2024; Watson et al., 2022).

Between-study heterogeneity was assessed using Cochrane’s *Q* and the *I*^2^ statistic expressed as percentages (25% = low heterogeneity, 50% = moderate heterogeneity, and 75% = high heterogeneity) (Higgins & Thompson, 2002; Higgins, Thompson, Deeks, & Altman, 2003).

To assess the presence of outliers in a sensitivity analysis, we used the “find.outliers” function from the “dmetar” R package when heterogeneity was above 50% (Harrer, Cuijpers, Furukawa, & Ebert, 2019). Outlying studies were removed for the patient group and for the control group separately, and the meta-analyses were conducted without the identified studies. Publication bias was assessed for each domain using the Egger’s test and visual inspection of funnel plots when the number of studies was >10 (Egger, Smith, Schneider, & Minder, 1997).

### Moderator analysis

We conducted meta-regressions for age, sex (percent male), and diagnosis (percent schizophrenia) for the within-subject change over time in the patient sample and for each domain. If a study reported age- or sex-adjusted cognitive data, those were excluded from the age and sex meta-regressions. In addition, we conducted subgroup analyses comparing studies with follow-ups of 5–9 years to studies with follow-ups of 10 years or more, and a subgroup analysis comparing FEP studies to SSDs and enduring schizophrenia studies. We also conducted a subgroup analysis comparing studies that used single cognitive tests for a given domain versus studies that used multiple tests. Subgroup analyses were conducted when the number of studies was equal to or greater than 10.

### FEP only meta-analysis

In addition to the main meta-analysis, including both SSD and FEP studies, and using the same methodology, we conducted an additional meta-analysis limited to FEP studies with a first cognitive assessment conducted at baseline. To further parse out heterogeneity, we also conducted moderator analyses by diagnosis (schizophrenia and schizoaffective disorder versus other) and by percent attrition.

## Results

### Search results and descriptives

**Figure 1.**
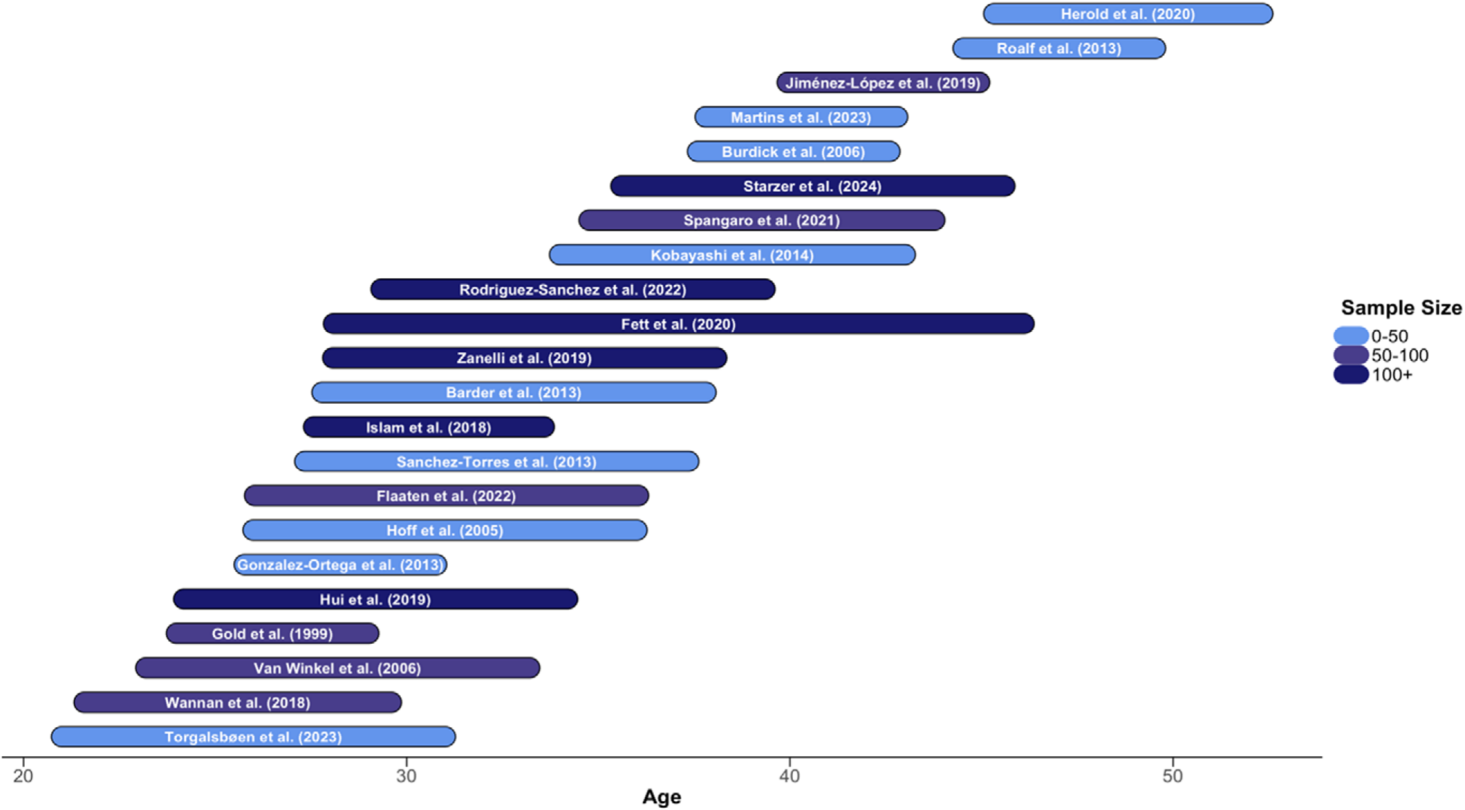
Included studies as a function of sample size, mean age at first assessment, and duration of follow-up. This figure displays the wide range of ages captured in the meta-analysis. Each bar represents a study and starts at the average age at first assessment. The length represents the duration of follow-up. The bars are color-coded based on sample size. Two studies, Hedman et al. (2012) and Stirling et al. (2003), are not included due to unreported age data.

The flowchart of included studies is outlined in Supplementary eFigure 1, and the characteristics of the included studies are presented in Table 1. The database search yielded 24 included studies (Supplementary eAppendix 1). They consisted of 2,633 patients and 1,019 controls, and 64.8% of the patients were male, and 35.2% were female. The average age of patients and controls at the first time point was 27.7 and 27.4 years, respectively. The average follow-up period of included studies was 8.46 years, and 61% of patients had a diagnosis of schizophrenia. Figure 1 illustrates the included studies based on sample size, age at first assessment, and duration of follow-up.

**Table 1.** Characteristics of included studies^a^

Study	Sample size, no. (tp1)	Population	Age (tp1), mean (SD)	Sex ratio, M:F	Follow-up period (years)	Domains assessed	Main finding
Barder et al. (2013)	43	FEP	27.8 (8.8)	22:21	10	VM, RP, VF	Stability
Burdick et al. (2006)	16	SSD	37.6 (4.9)	NA^d^	5	VF, RP, SP, VM	Stability
Fett et al. (2020)	195	FEP	28.1 (8.4)	112:83	18	SP, VM, ViM, RP, VF	Decline
Flaaten et al. (2022)^b^	75	FEP	26 (7.7)	41:34	10	SP, WM, VM, RP, VF	Stability
Gold, Arndt, Nopoulos, O’Leary, and Andreasen (1999)	54	FEP	24 (4.7)	41:13	5	Global (IQ), RP, ViM, VM, VF	Improvement
Gonzalez-Ortega et al. (2013)	26	FEP	25.8 (7.4)	15:11	5	RP, WM, VM, VF, SP	Improvement
Hedman et al. (2012)	9	Enduring	NA^d^	6:3	5	Global (IQ)	Stability
Herold, Duval, and Schröder (2021)	21	Enduring	45.3 (8.7)	15:6	7	SP, WM, VM, VF	Decline
Hoff, Svetina, Shields, Stewart, and DeLisi (2005)	21	FEP	26 (6.1)	15:6	10	RP, SP, VM	Stability
Hui et al. (2019)^b^	142	FEP	24.19 (6.4)	65:77	10	SP, RP, WM, VM, ViM, VF	NA^c^
Islam et al. (2018)^b^	1119	SSD	27.6 (7.9)	852:267	6	SP; ATT, VM	Stability
Jiménez-López et al. (2019)	79	SSD	39.9 (10.1)	39:40	5	Global, SP, VF, WM, VM, ViM, RP, ATT	Stability
Kobayashi et al. (2014)	36	SSD	34 (NA)^d^	21:15	9	VM, ViM, RP	Stability
Martins et al. (2023)	27	SSD	37.8 (11.2)	17:10	5	VM	Stability
Roalf et al. (2013)	34	Enduring	44.5 (8.5)	19:15	5	SP, RP, ATT, VM, ViM	Stability in accuracy; decline in speed
Rodríguez-Sánchez et al. (2022)	140	FEP	29.34 (8.1)	77:63	10	Global, SP, ATT, WM, VM, ViM, RP	Stability
Sánchez-Torres et al. (2013)	34	SSD	27.4 (5.6)	24:10	10	RP	Stability
Starzer et al. (2024)^b^	129	SSD	35.6 (NA)^d^	62:67	10	SP, WM, VM, RP, VF	Stability (subgroup with decline)
Spangaro et al. (2021)	93	SSD	34.8 (11.1)	62:31	9	Global, SP, WM, VM, RP, VF	Decline in treatment resistant, improvement in nontreatment resistant
Stirling et al. (2003)	37	FEP	NA^d^	NA^d^	10	VM, ViM, RP, VF	Decline in visual memory; stability in reasoning and problem-solving;
Torgalsbøen et al. (2023)^b^	28	FEP	21 (2.6)	17:11	10	Global, SP, ATT, WM, VM, ViM, RP	Improvement
van Winkel et al. (2006)	80	FEP	23.2 (4)	57:23	10	Global (IQ)	Stability
Wannan et al. (2018)	95	FEP	21.6 (3.4)	67:28	8	Global, VM, ViM	Decline in visual memory; stability in verbal memory
Zanelli et al. (2019)	106	FEP	28.1 (9.5)	63:43	10	Global (IQ), VM, ViM, SP, RP, VF	Decline

Abbreviations: ATT, attention; FEP, first-episode psychosis; IQ, intelligence quotient; NA, not applicable; RP, reasoning and problem-solving; SP, speed of processing; SSD, schizophrenia spectrum disorder; VF, verbal fluency; VM, verbal memory; ViM, visual memory; WM, working memory.

a All studies included in the meta-analysis.

b Data provided by authors.

c Study did not evaluate cognition directly.

d Data not available/provided.

### Baseline differences

Results of the patient-control meta-analyses at baseline are presented in Supplementary eTable 5. Patients presented with large cognitive impairments in all domains, ranging from *g* = −0.73; 95% CI = −0.93, −0.54 for working memory to *g* = −1.17; 95% CI = −1.42, −0.92 for the speed of processing.

### Change in cognition over time

**Figure 2.**
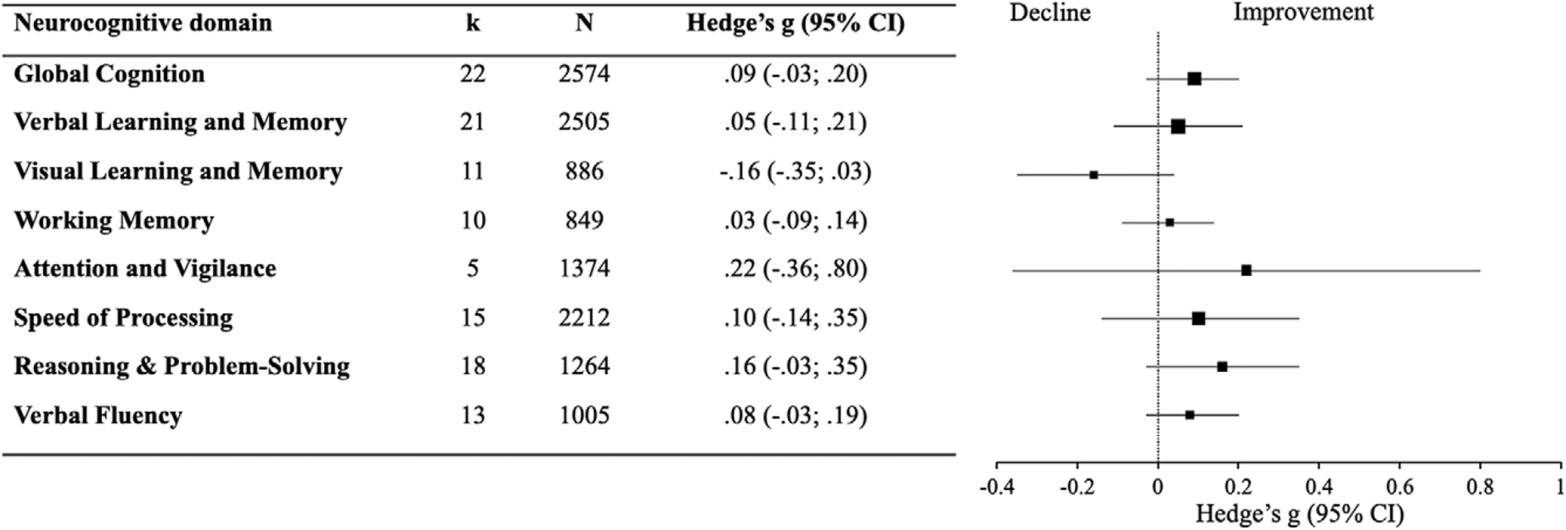
Forest plot for the change in cognition over time in patients. Abbreviations: *k*, number of studies; *N*, sample size. The large confidence interval for attention and vigilance can be explained by an outlying study and the small number of total studies (forest plots showing individual studies are presented in Supplementary eFigure 2).

Results for the change over time in all cognitive measures for patients and controls, and the forest plot for patients are presented in Table 2 and Figure 2, respectively. Forest plots for the individual domains for patients and controls are presented in Supplementary eFigures 2 and 3, respectively. Cognition remained stable in patients across all domains, with no significant change in global cognition (*g* = 0.09; 95% CI = −0.03, 0.20), verbal learning and memory (*g* = 0.05; 95% CI = −0.11, 0.21), visual learning and memory (*g* = −0.16; 95% CI = −0.35, 0.03), working memory (*g* = 0.03; 95% CI = −0.09, 0.14), attention (*g* = 0.22; 95% CI = −0.36, 0.80), the speed of processing (*g* = 0.10; 95% CI = −0.14, 0.35), reasoning and problem-solving (*g* = 0.16; 95% CI = −0.03, 0.35), and verbal fluency (*g* = 0.08; 95% CI = −0.03, 0.19). By contrast, controls significantly improved in global cognition (*g* = 0.17; 95% CI = 0.02; 0.33) and reasoning and problem-solving (*g* = 0.35; 95% CI = 0.18, 0.52), and the *Q*-test revealed a significant difference between patients and controls for these domains. Controls significantly improved in the speed of processing (*g* = 0.36; 95% CI = 0.01; 0.72), but did not differ significantly from patients (*Q* = 4.65, *p* = 0.10). Controls remained stable in working memory (*g* = 0.22; 95% CI = −0.04; 0.47), verbal learning and memory (*g =* 0.10; 95% CI = −0.25; 0.45), attention (*g* = 0.07; 95% CI = −0.36, 0.50), and visual learning and memory (*g =* −0.02; 95% CI = −0.42; 0.39) with no significant difference relative to patients. Heterogeneity ranged from moderate to high for all domains, and there was no evidence of publication bias (funnel plots presented in Supplementary eFigure 4). The results remained unchanged after varying the correlation coefficient (Supplementary eTables 6 and 7).

**Table 2.** Meta-analyses of change in cognition over time for patients and controls

Neurocognitive domain	k	N	Hedge’s g (95% CI)	Z	P	Q	*Q*(*p*)	*I*^2^%	Bias(*p*)	QM(*p*)
Global cognition										
Patients	22	2574	0.09 (−0.03; 0.20)	1.50	0.13	49.65	0.0004	59.49	0.42	6.62 (0.04)^a^
Controls	10	1001	0.17 (0.02; 0.33)	2.15	0.03	14.55	0.10	41.01	0.16	
Verbal learning and memory										
Patients	21	2505	0.05 (−0.11; 0.21)	0.62	0.53	91.70	<0.0001	81.92	0.24	0.80 (0.67)
Controls	9	962	0.10 (−0.25; 0.45)	0.55	0.58	51.17	<0.0001	88.19	–	
Visual learning and memory										
Patients	11	886	−0.16 (−0.35; 0.03)	−1.67	0.09	30.12	0.0008	69.66	0.12	1.95 (0.38)
Controls	6	249	−0.02 (−0.42; 0.39)	−0.05	0.94	19.72	0.001	78.46	–	
Working memory										
Patients	10	849	0.03 (−0.09; 0.14)	0.44	0.66	11.08	0.27	24.61	0.55	3.61 (0.16)
Controls	4	195	0.22 (−0.04; 0.47)	1.66	0.10	5.18	0.16	31.94	–	
Attention and vigilance										
Patients	5	1374	0.22 (−0.36; 0.80)	0.74	0.46	25.70	<0.0001	94.66	–	0.77 (0.68)
Controls	5	711	0.07 (−0.36; 0.50)	0.33	0.74	16.78	0.0021	83.47	–	
Speed of processing										
Patients	15	2212	0.10 (−0.14; 0.35)	0.84	0.40	107.40	<0.0001	90.88	0.49	4.65 (0.10)
Controls	7	832	0.36 (0.01; 0.72)	2.03	0.04	26.11	0.0002	84.41	–	
Reasoning and problem-solving										
Patients	18	1264	0.16 (−0.03; 0.35)	1.64	0.10	68.16	<0.0001	80.14	0.07	9.12 (0.01)^a^
Controls	8	324	0.35 (0.18; 0.52)	4.01	<0.0001	9.64	0.21	12.55	–	
Verbal fluency										
Patients	13	1005	0.08 (−0.03; 0.19)	1.46	.14	18.85	0.09	28.38	0.84	–
Controls^b^	–		–	–	–	–	–	–	–	–

*Note: Q* represents the Cochran’s *Q* value as a measure of between-study heterogeneity. *I*^2^ represents the percentage of variation between studies explained by heterogeneity (25% low heterogeneity, 50% moderate heterogeneity, and 75% high heterogeneity). Bias represents the *p*-value of the Egger’s test used to measure publication bias (reported when *k* = 10 or more). The QM(*p*) is the result of the subgroup analysis comparing the change in cognition between patients and controls.

Abbreviations: *k*, number of studies, *N*, sample size.

a A significant difference between patients and controls (*p* < 0.05).

b Not enough studies assessing verbal fluency in controls (*k* = 2).

Following outlier exclusion, the results were maintained for all domains except for (1) visual learning and memory, where a decline of small magnitude was observed in patients (*g* = −0.24; 95% CI = −0.39, −0.09), and significantly differed from controls who showed no change (*Q* = 11.92, *p* = 0.003), and (2) the speed of processing, where controls no longer significantly improved (*g* = 0.18; 95% CI = −0.01, 0.38) (Supplementary eTables 8 and 9 for a list of outlying studies and results without outliers, respectively).

The meta-analyses comparing change scores over time in studies, including both patients and controls, demonstrated no significant differences in the rate of change over time for any domain (Supplementary eTable 10). However, after the exclusion of an outlier, a significant difference in the rate of change over time emerged for visual learning and memory (*g* = −0.40; 95% CI = −0.79, −0.01).

### Moderator results

Meta-regression results are presented in Supplementary eTable 11. Older age was associated with a smaller improvement in verbal learning and memory (*Q* = 5.25, *p* = 0.02) and verbal fluency performance (*Q* = 7.61, *p* = 0.01). There was no significant moderating effect of sex. However, a higher percentage of individuals diagnosed with schizophrenia were associated with a greater improvement in visual learning and memory (*Q* = 4.41, *p* = 0.04) and working memory performance (*Q* = 4.73, *p* = 0.03) (scatterplots presented in Supplementary eFigure 5). Subgroup analyses revealed no significant differences between studies with follow-ups of 5–9 years relative to those with follow-ups of 10+ years for any measure (Supplementary eTable 12). There was also no difference between FEP studies and SSDs/enduring schizophrenia studies for any measure (Supplementary eTable 13). However, a trend-level difference was found for verbal fluency (*Q* = 5.59, *p* = 0.06), whereby FEP patients did significantly improve (*g =* 0.16; 95% CI = 0.02, 0.30). Finally, there was no difference between studies using single tests and studies using multiple tests in any domain (Supplementary eTable 14).

### FEP-only meta-analysis and moderator results

The meta-analyses of FEP studies with a first cognitive assessment at baseline revealed a significant improvement of small magnitude in global cognition (*g =* 0.17; 95% CI = 0.01, 0.32) but a small-to-moderate improvement in controls (*g =* 0.37, 95% CI = 0.17, 0.57), and the difference between the two groups was significant (*Q* = 12.89, *p* = 0.002). There was also a significant improvement of small magnitude in verbal fluency in patients (*g =* 0.20; 95% CI 0.04, 0.36). No other significant improvements were observed for patients. However, controls significantly improved in verbal learning and memory (*g =* 0.48; 95% CI 0.28, 0.68), relative to patients (*Q* = 7.72, *p* = 0.02). They also significantly improved in reasoning and problem-solving (*g =* 0.49; 95% CI 0.10, 0.87) relative to patients (*Q* = 5.96, *p* = 0.05). It is important to note, however, that the number of studies was low, particularly for controls (results presented in Supplementary eTable 15). In addition, a primary outlier (Torgalsbøen et al., 2023) appeared to significantly increase the effect sizes for the speed of processing and reasoning and problem-solving. After its exclusion, the effect size for the speed of processing and reasoning and problem-solving for patients dropped to *g* = 0.15; 95% CI = −0.23, 0.52, and to *g* = 0.10; 95% CI = −0.06, 0.25, respectively.

The diagnosis moderator analysis revealed that studies with a higher percentage of schizophrenia and schizoaffective disorder was associated with a greater improvement in global cognition (*Q* = 7.52, *p* = 0.006) and visual learning and memory (*Q* = 5.01, *p* = 0.03). There was no significant moderating effect of attrition rate, but a trending association was observed for global cognition (*Q* = 2.89, *p* = 0.09) and the speed of processing (*Q* = 2.76, *p* = 0.096), such that a higher percentage of attrition was associated with a smaller rate of change in cognition over time.

## Discussion

In the present meta-analysis, we provided a comprehensive picture of the evolution of cognitive impairments over time in individuals with psychotic disorders. We observed that cognitive impairments remain stable in the long term across samples varying in age at first assessment, providing support for the neurodevelopmental – rather than neurodegenerative – view of psychotic disorders. The stable cognitive performance is largely consistent across measures and between patients and controls, with minor exceptions, such as improvements of small magnitude observed in controls in global cognition and reasoning and problem-solving. Although primarily stable, outlier exclusion revealed a decline limited to visual memory and of small magnitude in patients.

The stability in cognition is consistent with previous meta-analytic reviews with shorter follow-up periods that identified small improvements driven by practice effects (Bora & Murray, 2014; Watson et al., 2022). The absence of an improvement in the present study may be explained by the longer time periods, which may have reduced the influence of practice effects. Our main finding of stability is robust to several potential moderators, such as age, sex, the percentage of patients diagnosed with schizophrenia, and the follow-up period. In addition to corroborating previous meta-analyses with shorter timeframes, our results are aligned with studies showing stability of cognition in bipolar disorder (Burdick et al., 2006; Martins et al., 2023).

It can be argued that decline could be apparent if longer time periods are examined. Specifically, the only study with a follow-up beyond 10 years in our study – by Fett et al. (2020)) – observed a widespread decline in several domains over 18 years. This finding may suggest that follow-ups of 5–10 years are insufficient to capture the decline that occurs due to long periods of unemployment or social isolation. Nonetheless, two studies that could not be included in the present meta-analysis due to unreported data with follow-ups of 15 (Albus et al., 2020) and 20 years (Bonner-Jackson et al., 2010) observed cognitive stability in people with schizophrenia. Furthermore, one included study with 10-year follow-up looked specifically at the period from 10 to 20 years after psychosis onset and reported overall stability (Starzer et al., 2024). Furthermore, the study by Fett et al. (2020) emerged as an outlier in the global cognition and reasoning and problem-solving meta-analysis. Another notable outlier was the 10-year follow-up by Torgalsbøen et al. (2023), which observed large improvements in patients that may be partially explained by repeated testing and practice effects (nine assessments over 10 years). However, the authors also point out that their sample is younger than those in other studies, which may also account for the higher rates of improvement.

When examining FEP studies only with a first cognitive assessment at baseline, the overall finding of stability is maintained for most domains, but a modest improvement in global cognition and verbal fluency emerges. However, the improvement in verbal fluency could not be compared to controls due to the small number of studies. Similarly, the number of FEP studies for all domains was quite low, so these results should be interpreted with caution.

Nonetheless, stability over time does not suggest that cognitive impairments are not amenable to change. When thinking specifically about FEP – which may represent an essential window for intervention (Dazzan et al., 2015) – targeting cognitive impairments early in the course of illness, through therapeutic approaches such as cognitive remediation, confers improvements that are sustained in the long term (Lepage et al., 2024). One meta-analysis of cognitive remediation for schizophrenia observed improvements in global cognition of small to moderate effect (Vita et al., 2021), and another found that this improvement was maintained beyond the posttreatment assessment (Vita, Barlati, et al., 2024). Interestingly, a novel conceptualization of cognitive impairment as primary or secondary to schizophrenia may inform treatment selection (Vita, Nibbio, & Barlati, 2024). For instance, primary impairments arising from the neurobiological sequelae of schizophrenia may improve through cognitive remediation, whereas secondary impairments arising from secondary causes, such as negative or depressive symptoms, may improve by targeting the implicated secondary symptoms.

The modest decline in visual memory following outlier exclusion may be indicative of possible deterioration in the long term. A recent meta-analysis in FEP with a median follow-up of 2 years identified improvements over time in all 13 domains examined, except for visual memory (Catalan et al., 2024). They further observed a moderating effect of the follow-up period, with longer follow-ups being associated with declines in visual memory and verbal memory. The authors pointed to the relationship between the visual system and the later transition to psychosis as a possible explanation (Diamond, Silverstein, & Keane, 2022). Interestingly, another similar and more recent meta-analysis evaluating changes in cognition over time, but with shorter follow-up periods, replicated this finding (Ding et al., 2024). Perhaps the visual system shows a particular vulnerability in psychotic disorders and is thus prone to further deterioration over time. A recent study by Zhang et al. (2024) observed that individuals at clinical high risk who converted to psychosis displayed atypical eye movement patterns that remained stable up to a year following conversion.

### Limitations and future directions

The present meta-analysis evaluated the change in cognition over time in samples of individuals with psychotic disorders as a whole without capturing within-subject variability or specific subgroups prone to decline. In several studies, subgroups that declined were identified (Albus et al., 2020; Rodríguez-Sánchez et al., 2022; Starzer et al., 2024). Starzer et al. (2024) reported that around a third of the sample declined, and Albus et al. (2020) observed a decline in individuals with deficit schizophrenia. Future work should employ data-driven approaches to identify trajectories of cognition and predictors of cognitive decline or improvement.

In addition, averaging effect sizes to provide a composite score for each domain may not capture differences in specific domain subprocesses. Still, no differences between patients and controls were observed in a separate meta-analysis that included test-specific analyses (Watson et al., 2022).

Another limitation is the moderate-to-high heterogeneity observed for most domains, which may be explained by the variety of tests used to evaluate a single domain. The lowest heterogeneity was observed for verbal fluency, which was evaluated using the same test in most studies. Another meta-analysis from our group observed a similarly low heterogeneity for verbal fluency (Khalil et al., 2022). In addition, outlier exclusion reduced heterogeneity significantly but did not alter our results.

## Conclusions

The large cognitive impairments observed before and following psychosis onset remain stable in the long term, consistent with the neurodevelopmental view of psychotic disorders. However, visual memory is one domain that may show a decline of small magnitude in the long term. Future studies should focus on identifying subgroups susceptible to cognitive decline.

## Supporting information

Ghanem et al. supplementary material Ghanem et al. supplementary material

## Supplementary material

The supplementary material for this article can be found at http://doi.org/10.1017/S0033291725100627.
