# Supplementary material for: Cognitive change 5+ years since the onset of a psychotic disorder: A systematic review and meta-analysis
Source: Psychol Med. 2025 Jul 16;55:e198. doi: 10.1017/S0033291725100627 (PMC12315663; doi:10.1017/S0033291725100627)
Supplement: Ghanem et al. supplementary material [file S0033291725100627sup001.docx]

**Cognitive Change 5+ Years Since the Onset of a Psychotic Disorder: A Systematic Review and Meta-Analysis.**

**eTable 1.** PRISMA Checklist

**eTable 2.** Search Terminology for the Systematic Review and Meta-Analysis

**eTable 3.** Quality Assessment of Each Included Study with the Mixed Methods Appraisal Tool (MMAT)

**eTable 4.** Cognitive Test Allocations for Each Cognitive Domain

**eFigure 1.** PRISMA Flowchart of Included Studies

**eAppendix 1.** References of Included Studies

**eTable 5.** Meta-Analyses of Baseline Differences in Cognition Between Patients and Controls

**eFigure 2.** Forest Plots for the Within-Subject Change in Patients for Each Domain

**eFigure 3.** Forest Plots for the Within-Subject Change in Controls for Each Domain

**eFigure 4.** Funnel Plots Per Domain for the Within-Subject Change Over Time in Patients

**eTable 6.** Main Results with Correlation Coefficient of rho=.58

**eTable 7.** Main Results with Correlation Coefficient of rho=.70

**eTable 8.** Table of Outliers

**eTable 9.** Main Results Excluding Outliers

**eTable 10.** Meta-Analyses Results of the Comparison of Change Scores Over Time in Studies Including Both Patients and Controls

**eTable 11.** Meta-Regression Results for Age, Sex, and Diagnosis

**eFigure 5.** Scatterplots of the Percent Schizophrenia Meta-Regression for Visual Memory and Working Memory

**eTable 12.** Results of the Follow-Up Period Subgroup Analysis

**eTable 13.** Results of the Subgroup Analysis Comparing FEP studies to Other Studies

**eTable 14.** Results of the Subgroup Analysis Comparing Single Test vs. Multiple Test Studies

**eTable 15.** Meta-Analyses Results of FEP Studies with Cognition Assessed at Baseline


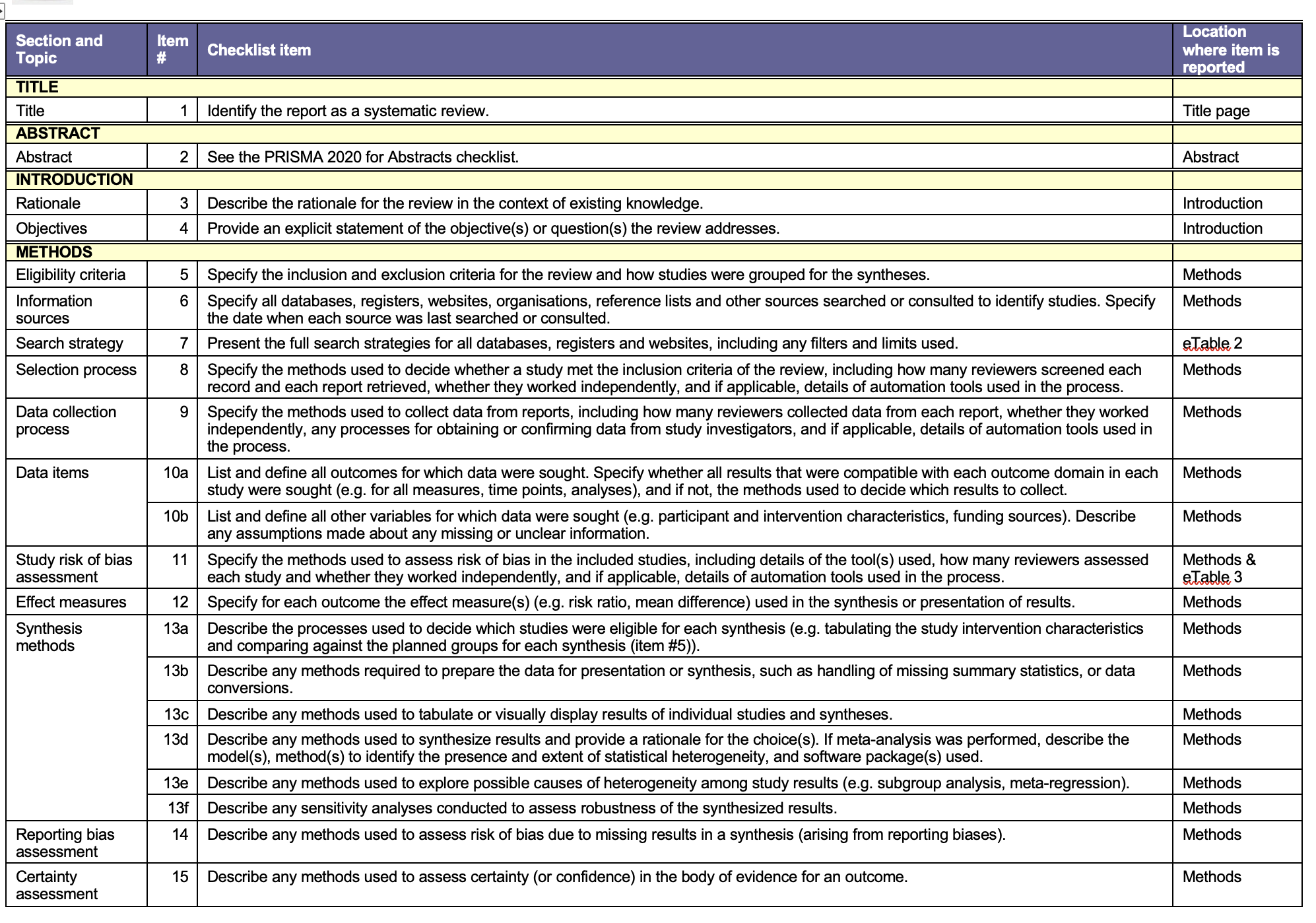
**eTable 1. PRISMA Checklist**


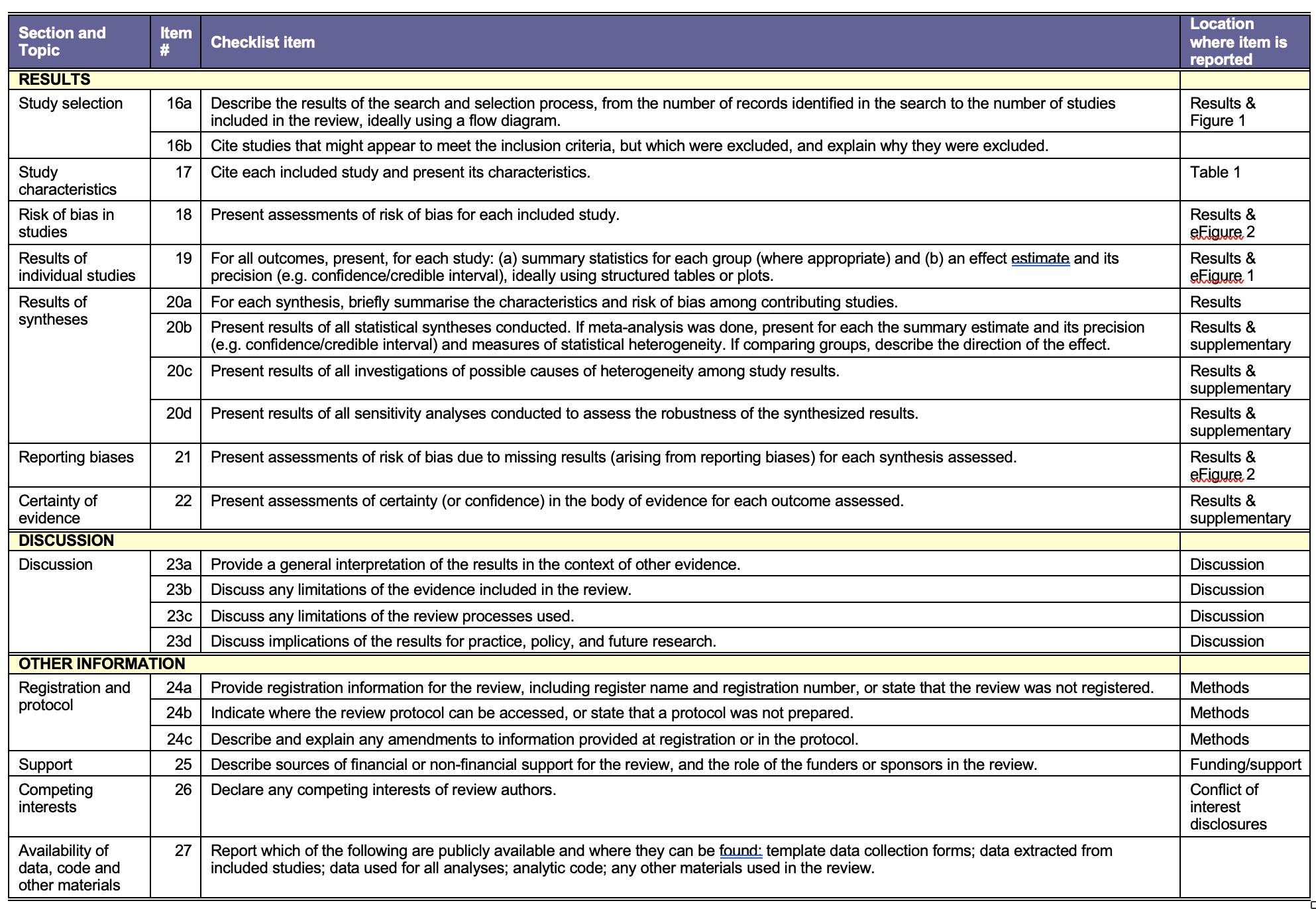


**eTable 2. Search Terminology for the Systematic Review and Meta-Analysis**

| **Database** | **Search Terminology** |
| --- | --- |
| MEDLINE | 1 Psychotic Disorders/  2 Schizophrenia/  3 first episode psychosis.mp.  4 Schizophreni*.mp.  5 1 or 2 or 3 or 4  6 Cognition/  7 Processing Speed/  8 Memory, Episodic/  9 verbal memory.mp.  10 Intelligence/  11 Learning/  12 Attention/  13 Longitudinal Studies/  14 longitudinal.mp.  15 Prospective Studies/  16 prospective.mp.  17 Executive Function/  18 reasoning.mp.  19 visual memory.mp.  20 Verbal Learning/  21 Neuropsychology/  22 neurocognit*.mp.  23 6 or 7 or 8 or 9 or 10 or 11 or 12 or 17 or 18 or 19 or 20 or 21 or 22  24 long-term.mp.  25 13 or 14 or 15 or 16 or 24  26 5 and 23 and 25 |
| PsycINFO | 1 first episode psychosis.mp.  2 Schizophreni*.mp.  3 Psychosis/  4 Schizophrenia/  5 cognition/  6 cognitive processing speed/  7 neurocognit*.mp.  8 Memory/  9 Attention/  10 Verbal Memory/  11 intelligence/  12 visual memory/  13 verbal learning/  14 neurocognition/  15 executive function/  16 reasoning/  17 Longitudinal Studies/  18 longitudinal.mp.  19 Prospective Studies/  20 prospective.mp.  21 cohort.mp.  22 long-term.mp.  23 neuropsychology/  24 1 or 2 or 3 or 4  25 5 or 6 or 7 or 8 or 9 or 10 or 11 or 12 or 13 or 14 or 15 or 16 or 23  26 17 or 18 or 19 or 20 or 21 or 22  27 24 and 25 and 26 |
| SCOPUS | ( TITLE-ABS ( schizophreni* OR psychosis ) ) AND ( TITLE-ABS ( neurocognit* OR neuropsycholog* OR "verbal memory" OR "visual memory" OR "processing speed" OR attention OR intelligence OR "executive function" OR reasoning ) ) AND ( TITLE-ABS ( longitudinal OR long-term OR prospective OR cohort ) ) |

**eTable 3. Quality Assessment of Each Included Study with the Mixed Methods Appraisal Tool (MMAT)**

| **Study** | **Screening Questions** | | | **Quantitative Descriptive Studies** | | | | **MMAT Score** | **Comments** |
| --- | --- | --- | --- | --- | --- | --- | --- | --- | --- |
|  | S1. Are there clear research questions | S2. Do the collected data allow to address the research questions? | 1. Is the sampling strategy relevant to address the research question? | 2. Is the sample representative of the target population? | 3. Are the measurements appropriate | 4. Is the risk of nonresponse bias low? | 5. Is the statistical analysis appropriate to answer the research question? |  |  |
| Rodriguez-Sanchez et al. (2022) | Yes | Yes | Yes | Yes | Yes | Yes | Yes | **5** | Compared individuals who completed the cognitive battery to those who did not. |
| Sanchez-Torres et al. (2013) | Yes | Yes | Yes | Yes | Yes | Yes | Yes | **5** |  |
| Fett et al. (2020) | Yes | Yes | Yes | Yes | Yes | Yes | Yes | **5** |  |
| Barder et al. (2013) | Yes | Yes | Yes | Yes | Yes | Yes | Yes | **5** |  |
| Torgalsbøen et al. (2023) | Yes | Yes | Yes | Yes | Yes | Yes | Yes | **5** |  |
| Stirling et al. (2003) | Yes | Yes | Yes | Yes | Yes | Yes | Yes | **5** |  |
| Hoff et al. (2005) | Yes | Yes | Yes | Yes | Yes | Yes | Yes | **5** |  |
| Van Winkel et al. (2006) | Yes | Yes | Yes | Yes | Yes | Yes | Yes | **5** |  |
| Flaaten et al. (2022) | Yes | Yes | Yes | Yes | Yes | No | Yes | **4** |  |
| **Study** | **Screening Questions** | | | **Quantitative Descriptive Studies** | | | | **MMAT Score** | **Comments** |
|  | S1. Are there clear research questions | S2. Do the collected data allow to address the research questions? | 1. Is the sampling strategy relevant to address the research question? | 2. Is the sample representative of the target population? | 3. Are the measurements appropriate | 4. Is the risk of nonresponse bias low? | 5. Is the statistical analysis appropriate to answer the research question? |  |  |
| Starzer et al. (2024) | Yes | Yes | Yes | Yes | Yes | No | Yes | **4** |  |
| Islam et al. (2018) | Yes | Yes | Yes | Yes | Yes | Yes | Yes | **5** | Missing data handled |
| Herold et al. (2020) | Yes | Yes | Yes | Yes | Yes | No | Yes | **4** | No information on number of people initially assessed. Did not account for 7 people in nursing home. |
| Kobayashi et al. (2014) | Yes | Yes | Yes | Yes | Yes | Yes | Yes | **5** |  |
| Spangaro et al. (2021) | Yes | Yes | Yes | Yes | Yes | No | Yes | **4** | Number of missing people initially assessed |
| Wannan et al. (2018) | Yes | Yes | Yes | Yes | Yes | No | Yes | **4** | Accounted for missing data from follow up participants, but not for those who could not be followed up |
| Gold et al. (1999) | Yes | Yes | Yes | Yes | Yes | No | Yes | **4** |  |
| Martins et al. (2023) | Yes | Yes | Yes | Yes | Yes | Yes | Yes | **5** |  |
| Burdick et al. (2006) | Yes | Yes | Yes | Yes | Yes | Yes | Yes | **5** |  |
|  | | | | | | | | | |
| **Study** | **Screening Questions** | | | **Quantitative Descriptive Studies** | | | | **MMAT Score** | **Comments** |
|  | S1. Are there clear research questions | S2. Do the collected data allow to address the research questions? | 1. Is the sampling strategy relevant to address the research question? | 2. Is the sample representative of the target population? | 3. Are the measurements appropriate | 4. Is the risk of nonresponse bias low? | 5. Is the statistical analysis appropriate to answer the research question? |  |  |
| Hedman et al. (2012) | Yes | Yes | Yes | No | Yes | Yes | Yes | **4** |  |
| Gonzalez-Ortega et al. (2013) | Yes | Yes | Yes | Yes | Yes | Yes | Yes | **5** |  |
| Zanelli et al. (2019) | Yes | Yes | Yes | Yes | Yes | Yes | Yes | **5** |  |
| Roalf et al. (2013) | Yes | Yes | Yes | Yes | Yes | Yes | Yes | **5** |  |
| Jiménez-López et al. (2019) | Yes | Yes | Yes | Yes | Yes | Yes | Yes | **5** |  |
| Hui et al. (2019) | Yes | Yes | Yes | Yes | Yes | Yes | Yes | **5** |  |

*Note.* Studies were rated based on our outcome of interest and on the individual studies included in the meta-analysis and not on the original investigation on which they are based. For example, a study may have been rated as quantitative descriptive even if the original sample was part of a randomized controlled trial. This was done because our outcome of interest was not a specific intervention or exposure but rather the change in cognition over time. Methodological quality is based on 5 questions (a “yes” response would count as 1 point, a “no” or “can’t tell” response would count as 0). The risk of nonresponse bias was assessed as follows: a rating of 0 was given if there were statistically significant differences between respondents and nonrespondents on 2 variables or more. A rating of 1 was given if missing data was handled (imputation, models that are sensitive to missing values). A rating of 0 was given If more than 20% of the sample was missing at the follow-up.

**eTable 4. Cognitive Test Allocation for Each Cognitive Domain**

| **Domain** | **Tests** |
| --- | --- |
| Verbal Learning and Memory | Weschler Memory Scale (WMS) logical memory I (immediate), WMS logical memory II (delayed), California Verbal Learning Test (CVLT) total immediate recall, CVLT delayed free recall, CVLT mean errors at recall, CVLT recognition, Rey Auditory Verbal Learning Test (RAVLT) immediate, RAVLT delayed, WMS Verbal paired associates I (immediate), WMS verbal paired associates II (delayed), Hopkins Verbal Learning Test Revised (HVLT-R), Warrington recognition memory tests for words (WRMTW), BACS verbal memory, World Learning Task (WLT) immediate, WLT delayed recall, PennCNB verbal memory (speed), PennCNB verbal memory (accuracy) |
| Visual Learning and Memory | WMS visual reproduction I (immediate), WMS visual reproduction II (delayed), Rey Complex Figure Test (immediate), Rey Complex Figure Test (delayed), Visual Patterns Test, Brief Visuospatial Memory test Revised (BVMT-R), Warrington recognition memory tests for words (WRMTF), Memory for Design test (MFD), Cambridge Neuropsychological Test Automated Battery (CANTAB) paired-associates learning (PAL) number of errors, PennCNB face memory (speed), PennCNB face memory (accuracy) |
| Working Memory | Digit Span total, backwards, forwards, Letter Number Sequencing (LNS), WMS Spatial Span, BACS digit sequencing |
| Attention and Vigilance | Continuous Performance Task (CPT) performance, CPT variance, CPT Identical pairs (CPT-IP), PennCNB attention (speed), PennCNB attention (accuracy) |
| Speed of Processing | Trail-Making Task A (TMT-A), Digit Symbol, Symbol Digit Modalities test (SDMT) written, SDMT oral, Brief Assessment of Cognition (BACS) Symbol Coding, D-KEFS Color-Word Interference Test (CWIT) (color naming), D-KEFS CWIT reading, Stroop word, Stroop color, PennCNB sensorimotor processing speed |
| Reasoning & Problem-Solving | Trail-Making Task B (TMT-B), TMT difference, Wisconsin Card Sorting Test (WCST) categories completed, WCST total errors, WCST perseverative errors, WCST conceptual level responses, WCST failure to maintain set, WCST attempts to first category, Stroop color word test, Stroop interference, mazes from the Neuropsychological Assessment Battery (NAB mazes), D-KEFS CWIT Interference, D-KEFS CWIT switching, BACS Tower of London, PennCNB abstraction and mental flexibility (speed), PennCNB abstraction and mental flexibility (accuracy) |
| Verbal Fluency | Controlled Oral Word Association Test (COWAT) (FAS fluency), semantic animal fluency, D-KEFS verbal fluency FAS, D-KEFS verbal fluency categories, D-KEFS verbal fluency switching, BACS fluency, MAE oral word association test score |

**eFigure 1. PRISMA flowchart of Included Studies**

**Identification of studies via other methods**

**Identification of studies via databases and registers**

Records identified from:

Snowballing (n =105)

Records removed *before screening*:

Duplicate records removed (n =1930)

Records identified from*:

MEDLINE (n = 1798)

SCOPUS (n = 2950)

PsycINFO (n= 1570)

Total (n = 6318)

**Identification**

Records screened

(n = 4392)

Records excluded**

(n = 4266)

Reports not retrieved

(n = 0 )

Reports sought for retrieval

(n = 3)

Reports not retrieved

(n =0)

Reports sought for retrieval

(n = 126)

**Screening**

Reports excluded: 102

Follow-up <5 years (n = 31)

Duplicate sample (n = 32)

Wrong patient population (n =17)

Wrong study design (n=11)

No baseline/follow-up cognitive assessment (n=6)

No standardized cognitive battery (n=2)

Not assessing MATRICS domains (n=1)

Reports excluded: 3

Follow-up <5 years (n = 2)

Wrong study design (n = 1)

Reports assessed for eligibility

(n = 3)

Reports assessed for eligibility

(n = 126)

**Included**

Studies included in meta-analysis

(n = 24)

**eAppendix 1. References of Included Studies**

Barder, H. E., Sundet, K., Rund, B. R., Evensen, J., Haahr, U., Ten Velden Hegelstad, W., Joa, I., Johannessen, J. O., Langeveld, J., & Larsen, T. K. (2013). Ten year neurocognitive trajectories in first-episode psychosis. *Frontiers in human neuroscience*, *7*, 643.

Burdick, K. E., Goldberg, J. F., Harrow, M., Faull, R. N., & Malhotra, A. K. (2006). Neurocognition as a stable endophenotype in bipolar disorder and schizophrenia. *The Journal of nervous and mental disease*, *194*(4), 255-260.

Fett, A.-K. J., Velthorst, E., Reichenberg, A., Ruggero, C. J., Callahan, J. L., Fochtmann, L. J., Carlson, G. A., Perlman, G., Bromet, E. J., & Kotov, R. (2020). Long-term changes in cognitive functioning in individuals with psychotic disorders: findings from the Suffolk County Mental Health Project. *JAMA psychiatry*, *77*(4), 387-396.

Flaaten, C. B., Melle, I., Bjella, T., Engen, M. J., Åsbø, G., Wold, K. F., Widing, L., Gardsjord, E., Sæther, L.-S., & Øie, M. G. (2022). Domain-specific cognitive course in schizophrenia: Group-and individual-level changes over 10 years. *Schizophrenia Research: Cognition*, *30*, 100263.

Gold, S., Arndt, S., Nopoulos, P., O’Leary, D. S., & Andreasen, N. C. (1999). Longitudinal study of cognitive function in first-episode and recent-onset schizophrenia. *American Journal of Psychiatry*, *156*(9), 1342-1348.

Gonzalez-Ortega, I., de Los Mozos, V., Echeburua, E., Mezo, M., Besga, A., de Azúa, S. R., Gonzalez-Pinto, A., Gutierrez, M., Zorrilla, I., & Gonzalez-Pinto, A. (2013). Working memory as a predictor of negative symptoms and functional outcome in first episode psychosis. *Psychiatry research*, *206*(1), 8-16.

Hedman, A., van Haren, N., van Baal, G., Brans, R., Hijman, R., Kahn, R., & Pol, H. H. (2012). Is there change in intelligence quotient in chronically ill schizophrenia patients? A longitudinal study in twins discordant for schizophrenia. *Psychological medicine*, *42*(12), 2535-2541.

Herold, C. J., Duval, C. Z., & Schröder, J. (2021). Neurological soft signs and cognition in the late course of chronic schizophrenia: a longitudinal study. *European Archives of Psychiatry and Clinical Neuroscience*, *271*(8), 1465-1473.

Hoff, A. L., Svetina, C., Shields, G., Stewart, J., & DeLisi, L. E. (2005). Ten year longitudinal study of neuropsychological functioning subsequent to a first episode of schizophrenia. *Schizophrenia Research*, *78*(1), 27-34.

Hui, C. L., Honer, W. G., Lee, E. H., Chang, W., Chan, S. K., Chen, E. S., Pang, E. P., Lui, S. S., Chung, D. W., & Yeung, W. (2019). Predicting first-episode psychosis patients who will never relapse over 10 years. *Psychological medicine*, *49*(13), 2206-2214.

Islam, M. A., Habtewold, T. D., van Es, F. D., Quee, P. J., van den Heuvel, E., Alizadeh, B., Bruggeman, R., Investigators, G., Bartels‐Velthuis, A. A., & van Beveren, N. J. (2018). Long‐term cognitive trajectories and heterogeneity in patients with schizophrenia and their unaffected siblings. *Acta Psychiatrica Scandinavica*, *138*(6), 591-604.

Jiménez-López, E., Sánchez-Morla, E. M., López-Villarreal, A., Aparicio, A. I., Martínez-Vizcaíno, V., Vieta, E., Rodriguez-Jimenez, R., & Santos, J. L. (2019). Neurocognition and functional outcome in patients with psychotic, non-psychotic bipolar I disorder, and schizophrenia. A five-year follow-up. *European Psychiatry*, *56*(1), 60-68.

Kobayashi, H., Isohanni, M., Jääskeläinen, E., Miettunen, J., Veijola, J., Haapea, M., Järvelin, M.-R., Jones, P. B., & Murray, G. K. (2014). Linking the developmental and degenerative theories of schizophrenia: association between infant development and adult cognitive decline. *Schizophrenia bulletin*, *40*(6), 1319-1327.

Martins, D. S., Hasse-Sousa, M., Reckziegel, R. d. F. X., Lapa, C. d. O., Petry-Perin, C., Britto, M. J., Remus, I. B., Gama, C. S., & Czepielewski, L. S. (2023). A five-year follow-up of the verbal memory performance of individuals with bipolar disorder and schizophrenia: evidence of unchanging deficits under treatment. *Cognitive neuropsychiatry*, *28*(1), 19-35.

Roalf, D. R., Gur, R. C., Almasy, L., Richard, J., Gallagher, R. S., Prasad, K., Wood, J., Pogue-Geile, M. F., Nimgaonkar, V. L., & Gur, R. E. (2013). Neurocognitive performance stability in a multiplex multigenerational study of schizophrenia. *Schizophrenia bulletin*, *39*(5), 1008-1017.

Rodríguez-Sánchez, J. M., Setién-Suero, E., Suárez-Pinilla, P., Van Son, J. M., Vázquez-Bourgon, J., López, P. G., Crespo-Facorro, B., & Ayesa-Arriola, R. (2022). Ten-year course of cognition in first-episode non-affective psychosis patients: PAFIP cohort. *Psychological medicine*, *52*(4), 770-779.

Sánchez-Torres, A. M., Basterra, V., Moreno-Izco, L., Rosa, A., Fañanás, L., Zarzuela, A., Peralta, V., & Cuesta, M. J. (2013). Executive functioning in schizophrenia spectrum disorder patients and their unaffected siblings: a ten-year follow-up study. *Schizophrenia Research*, *143*(2-3), 291-296.

Spangaro, M., Martini, F., Bechi, M., Buonocore, M., Agostoni, G., Cocchi, F., Sapienza, J., Bosia, M., & Cavallaro, R. (2021). Longitudinal course of cognition in schizophrenia: Does treatment resistance play a role? *Journal of Psychiatric Research*, *141*, 346-352.

Starzer, M., Hansen, H. G., Hjorthøj, C., Albert, N., Lewandowski, K. E., Glenthøj, L. B., & Nordentoft, M. (2024). 20-year neurocognitive development following a schizophrenia spectrum disorder and associations with symptom severity and functional outcomes. *Psychological medicine*, 1-11.

Stirling, J., White, C., Lewis, S., Hopkins, R., Tantam, D., Huddy, A., & Montague, L. (2003). Neurocognitive function and outcome in first-episode schizophrenia: a 10-year follow-up of an epidemiological cohort. *Schizophrenia Research*, *65*(2-3), 75-86.

Torgalsbøen, A.-K., Mohn, C., Larøi, F., Fu, S., & Czajkowski, N. (2023). A ten-year longitudinal repeated assessment study of cognitive improvement in patients with first-episode schizophrenia and healthy controls: The Oslo Schizophrenia Recovery (OSR) study. *Schizophrenia Research*, *260*, 92-98.

van Winkel, R., Myin-Germeys, I., Delespaul, P., Peuskens, J., De Hert, M., & van Os, J. (2006). Premorbid IQ as a predictor for the course of IQ in first onset patients with schizophrenia: a 10-year follow-up study. *Schizophrenia Research*, *88*(1-3), 47-54.

Wannan, C. M., Bartholomeusz, C. F., Cropley, V. L., Van Rheenen, T., Panayiotou, A., Brewer, W., Proffitt, T., Henry, L., Harris, M., & Velakoulis, D. (2018). Deterioration of visuospatial associative memory following a first psychotic episode: a long-term follow-up study. *Psychological medicine*, *48*(1), 132-141.

Zanelli, J., Mollon, J., Sandin, S., Morgan, C., Dazzan, P., Pilecka, I., Reis Marques, T., David, A. S., Morgan, K., & Fearon, P. (2019). Cognitive change in schizophrenia and other psychoses in the decade following the first episode. *American Journal of Psychiatry*, *176*(10), 811-819.

**eTable 5. Meta-Analyses of Baseline Differences in Cognition Between Patients and Controls**

| **Neurocognitive domain** | **k** | **N** | **Hedge’s g (95% CI)** | **Z** | **P** | **Q** | **Q(p)** | **I^2^%** | **Bias(p)** |
| --- | --- | --- | --- | --- | --- | --- | --- | --- | --- |
| Global Cognition | 10 | 2638 | -.86 (-.94, -.77) | -19.49 | <.0001^a^ | 9.67 | .38 | 0 | .88 |
| Verbal Learning and Memory | 10 | 2704 | -.94 (-1.12, -.75) | -9.92 | <.0001^a^ | 18.49 | .03 | 60.65 | .40 |
| Visual Learning and Memory | 6 | 612 | -.87 (-1.26, -.48) | -4.39 | <.0001^a^ | 21.49 | .001 | 77.04 | - |
| Working Memory | 4 | 515 | -.73 (-.93, -.54) | -7.29 | <.0001^a^ | 2.28 | .52 | 0 | - |
| Attention and Vigilance | 5 | 2085 | -.76 (-1.03, -.48) | -5.37 | <.0001^a^ | 10.81 | .03 | 62.86 | - |
| Speed of Processing | 7 | 2327 | -1.17 (-1.42, -.92) | -9.24 | <.0001^a^ | 14.22 | .03 | 66.25 | - |
| Reasoning & Problem-Solving | 8 | 764 | -.86 (-1.02, -.69) | -10.37 | <.0001^a^ | 4.29 | .75 | 0 | - |
| Verbal Fluency^b^ | 2 | 281 | - | - | - | - | - | - | - |

Abbreviations: k, number of studies, N, sample size. Q represents the Cochran’s Q value as a measure of between-study heterogeneity. *I^2^*  represents the percentage of variation between studies explained by heterogeneity (25% low heterogeneity, 50% moderate heterogeneity, 75% high heterogeneity). Bias represents the p-value of the egger’s test used to measure publication bias (reported when k=10 or more).

^a^Denotes a significant difference between patients and controls (p<.05).

^b^Not enough studies assessing verbal fluency in controls (k=2).

**eTable 6. Main Results with Correlation Coefficient of rho=.58**

| **Neurocognitive domain** | **k** | **N** | **Hedge’s g (95% CI)** | **Z** | **P** | **Q** | **Q(p)** | **I^2^%** | **Bias(p)** | **QM(p)** |
| --- | --- | --- | --- | --- | --- | --- | --- | --- | --- | --- |
| **Global Cognition** |  |  |  |  |  |  |  |  |  |  |
| Patients | 22 | 2574 | . 08 (-.02; .18) | 1.48 | .14 | 41.90 | .004 | 51.27 | .42 | 6.27 (.04)^a^ |
| Controls | 10 | 1001 | .15 (.01;.29) | 2.05 | .04 | 12.40 | .19 | 31.10 | .16 |  |
| **Verbal Learning and Memory** |  |  |  |  |  |  |  |  |  |  |
| Patients | 21 | 2505 | .05 (-.10; .20) | .63 | .53 | 77.73 | <.0001 | 78.20 | .24 | .80 (.67) |
| Controls | 9 | 962 | .09 (-.23; .41) | .55 | .58 | 43.90 | <.0001 | 86.10 | - |  |
| **Visual Learning and Memory** |  |  |  |  |  |  |  |  |  |  |
| Patients | 11 | 886 | -.15 (-.33; .02) | -1.74 | .08 | 25.41 | .005 | 63.55 | .15 | 2.17 (.34) |
| Controls | 6 | 249 | -.01 (-.38; .35) | -.06 | .95 | 16.80 | .005 | 73.90 | - |  |
| **Working Memory** |  |  |  |  |  |  |  |  |  |  |
| Patients | 10 | 849 | .02 (-.09; .13) | .38 | .70 | 9.30 | .41 | 13.01 | .55 | 3.41 (.18) |
| Controls | 4 | 195 | .19 (-.01; .39) | 1.90 | .06 | 4.43 | .22 | 0 | - |  |
| **Attention and Vigilance** |  |  |  |  |  |  |  |  |  |  |
| Patients | 5 | 1374 | .20 (-.33; .72) | .73 | .46 | 22.20 | .0002 | 93.46 | - | .76 (.69) |
| Controls | 5 | 711 | .07 (-.36; .50) | .33 | .74 | 16.78 | .0021 | 83.47 | - |  |
| **Speed of Processing** |  |  |  |  |  |  |  |  |  |  |
| Patients | 15 | 2212 | .10 (-.13; .32) | .85 | .40 | 91.13 | <.0001 | 88.84 | .49 | 4.64 (.10) |
| Controls | 7 | 832 | .33 (.01; .65) | 2.03 | .04 | 22.56 | .001 | 80.90 | - |  |
| **Reasoning & Problem-Solving** |  |  |  |  |  |  |  |  |  |  |
| Patients | 18 | 1264 | .14 (-.03; .31) | 1.62 | .11 | 58.90 | <.0001 | 75.14 | .07 | 9.16 (.01)^a^ |
| Controls | 8 | 324 | .32 (.17; .48) | 4.08 | <.0001 | 8.29 | .31 | 0 | - |  |
| **Verbal Fluency** |  |  |  |  |  |  |  |  |  |  |
| Patients | 13 | 1005 | .07 (-.02; .17) | 1.52 | .13 | 15.83 | .20 | 9.45 | .84 | - |
| Controls ^b^ | - |  | - | - | - | - | - | - | - | - |

Abbreviations: k, number of studies, N, sample size. Q represents the Cochran’s Q value as a measure of between-study heterogeneity. *I^2^*  represents the percentage of variation between studies explained by heterogeneity (25% low heterogeneity, 50% moderate heterogeneity, 75% high heterogeneity). Bias represents the p-value of the egger’s test used to measure publication bias (reported when k=10 or more). The QM(p) is the result of the subgroup analysis comparing the change in cognition between patients and controls.

^a^Denotes a significant difference between patients and controls (p<.05).

**eTable 7. Main Results with Correlation Coefficient of rho=.70**

| **Neurocognitive domain** | **k** | **N** | **Hedge’s g (95% CI)** | **Z** | **P** | **Q** | **Q(p)** | **I^2^%** | **Bias(p)** | **QM(p)** |
| --- | --- | --- | --- | --- | --- | --- | --- | --- | --- | --- |
| **Global Cognition** |  |  |  |  |  |  |  |  |  |  |
| Patients | 22 | 2574 | . 09 (-.03; .21) | 1.49 | .14 | 57.25 | <.0001 | 65.57 | .41 | 6.87 (.03)^a^ |
| Controls | 10 | 1001 | .19 (.02;.36) | 2.21 | .03 | 16.60 | .06 | 48.80 | .16 |  |
| **Verbal Learning and Memory** |  |  |  |  |  |  |  |  |  |  |
| Patients | 21 | 2505 | .05 (-.12; .23) | .62 | .54 | 105.35 | <.0001 | 84.52 | .24 | .79 (.67) |
| Controls | 9 | 962 | .11 (-.27; .48) | .55 | .58 | 58.10 | <.0001 | 89.69 | - |  |
| **Visual Learning and Memory** |  |  |  |  |  |  |  |  |  |  |
| Patients | 11 | 886 | -.17 (-.37; .03) | -1.63 | .10 | 34.74 | <.0001 | 73.94 | .12 | 1.82 (.40) |
| Controls | 6 | 249 | -.02 (-.46; .42) | -.09 | .93 | 22.54 | .0004 | 81.60 | - |  |
| **Working Memory** |  |  |  |  |  |  |  |  |  |  |
| Patients | 10 | 849 | .03 (-.10; .16) | .48 | .63 | 12.84 | .17 | 33.50 | .55 | 3.75 (.15) |
| Controls | 4 | 195 | .24 (-.05; .52) | 1.59 | .11 | 5.88 | .12 | 46.56 | - |  |
| **Attention and Vigilance** |  |  |  |  |  |  |  |  |  |  |
| Patients | 5 | 1374 | .24 (-.39; .86) | .74 | .46 | 28.98 | <.0001 | 95.46 | - | .78 (.68) |
| Controls | 5 | 711 | .08 (-.38; .55) | .35 | .73 | 19.18 | .0007 | 86.01 | - |  |
| **Speed of Processing** |  |  |  |  |  |  |  |  |  |  |
| Patients | 15 | 2212 | .11 (-.15; .37) | .84 | .40 | 123.17 | <.0001 | 92.30 | .48 | 4.66 (.10) |
| Controls | 7 | 832 | .40 (.01; .78) | 2.03 | .04 | 29.43 | <.0001 | 86.80 | - |  |
| **Reasoning & Problem-Solving** |  |  |  |  |  |  |  |  |  |  |
| Patients | 18 | 1264 | .18 (-.03; .38) | 1.65 | .10 | 76.88 | <.0001 | 83.48 | .06 | 9.07 (.01)^a^ |
| Controls | 8 | 324 | .38 (.18; .57) | 3.73 | .0002 | 10.91 | .14 | 30.13 | - |  |
| **Verbal Fluency** |  |  |  |  |  |  |  |  |  |  |
| Patients | 13 | 1005 | .09 (-.04 .21) | 1.40 | .16 | 21.83 | .04 | 41.56 | .84 | - |
| Controls ^b^ | - |  | - | - | - | - | - | - | - | - |

Abbreviations: k, number of studies, N, sample size. Q represents the Cochran’s Q value as a measure of between-study heterogeneity. *I^2^*  represents the percentage of variation between studies explained by heterogeneity (25% low heterogeneity, 50% moderate heterogeneity, 75% high heterogeneity). Bias represents the p-value of the egger’s test used to measure publication bias (reported when k=10 or more). The QM(p) is the result of the subgroup analysis comparing the change in cognition between patients and controls.

^a^Denotes a significant difference between patients and controls (p<.05).

**eFigure 2. Forest Plots for the Within-Subject Change in Patients for Each Domain.**

The estimate represents the effect size (Hedge’s g). RE model= random-effects model. The percentages represent the weights of each study.

1. Global Cognition

*
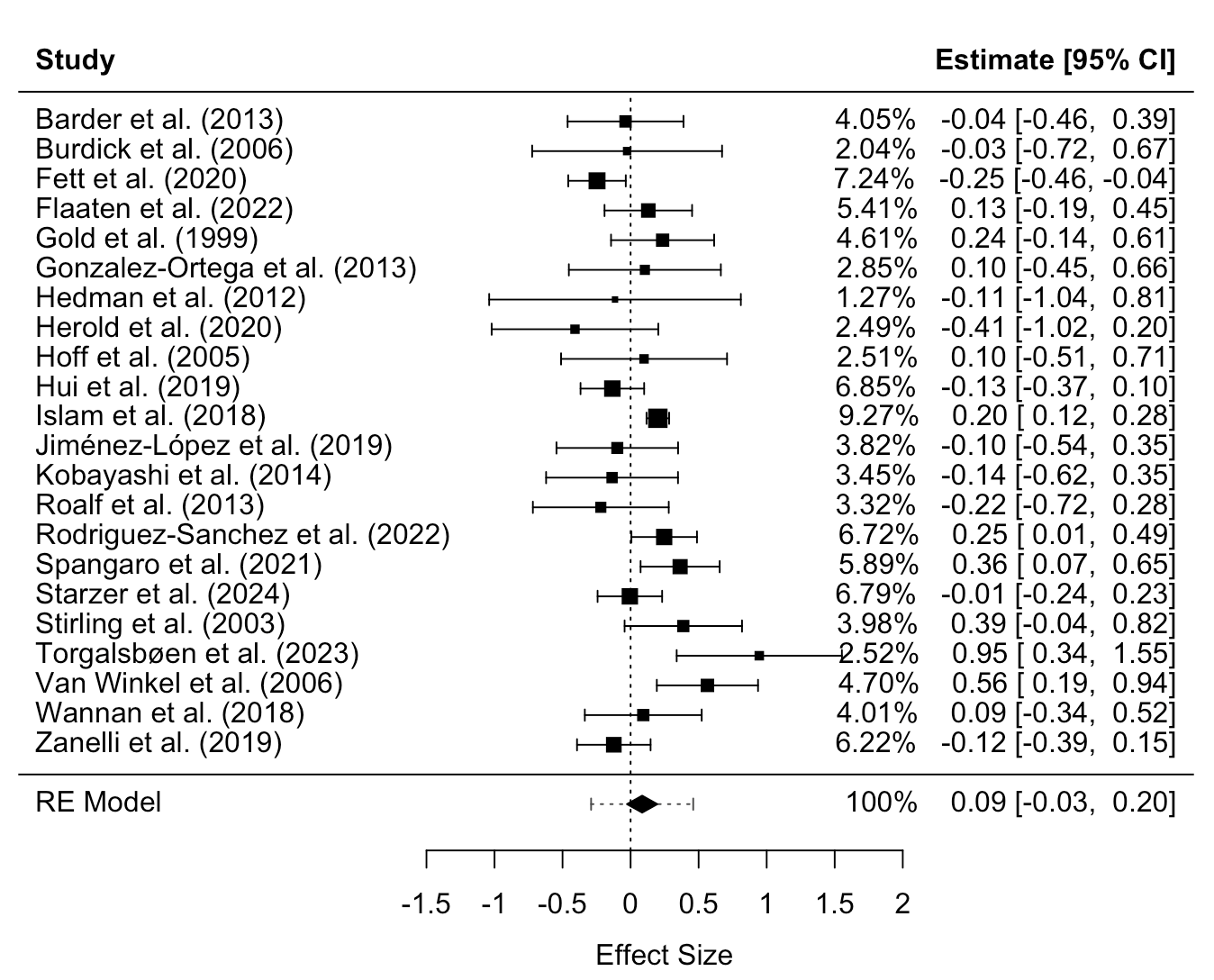
*

Heterogeneity: *I^2^* = 59.49%

1. Verbal Learning and Memory


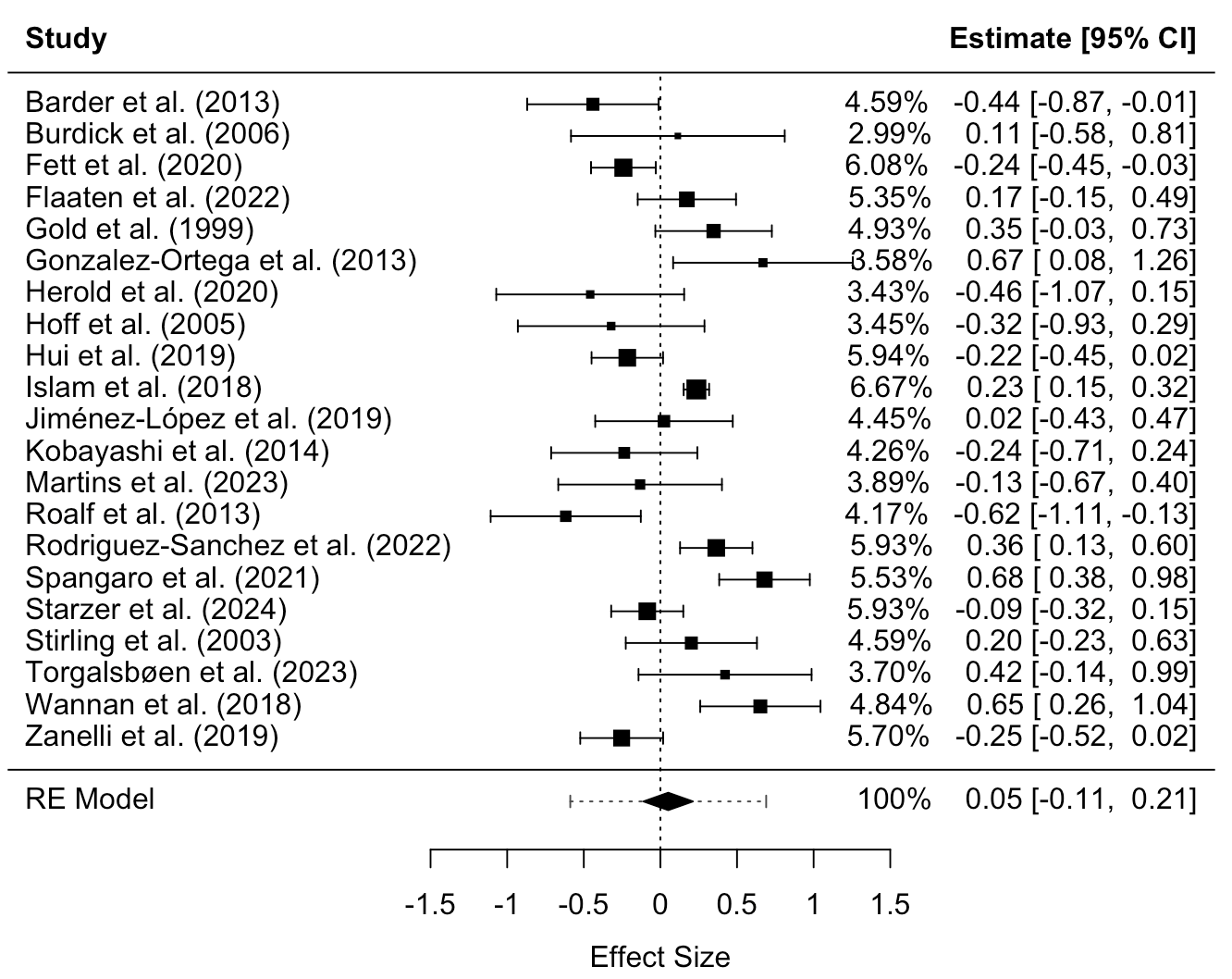


Heterogeneity: *I^2^* = 81.92%

1. Visual Learning and Memory


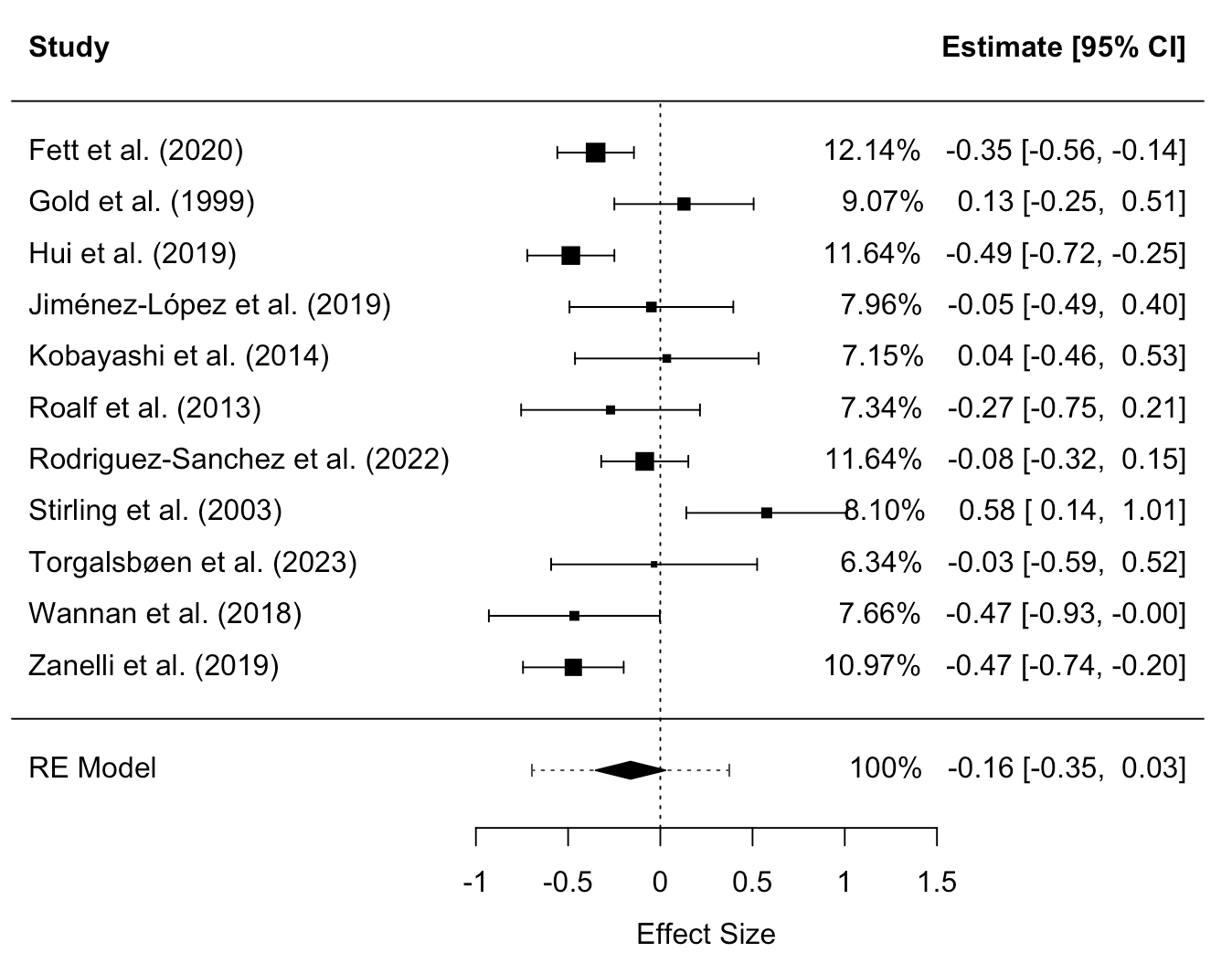


Heterogeneity: *I^2^* = 69.66%

1. Working Memory


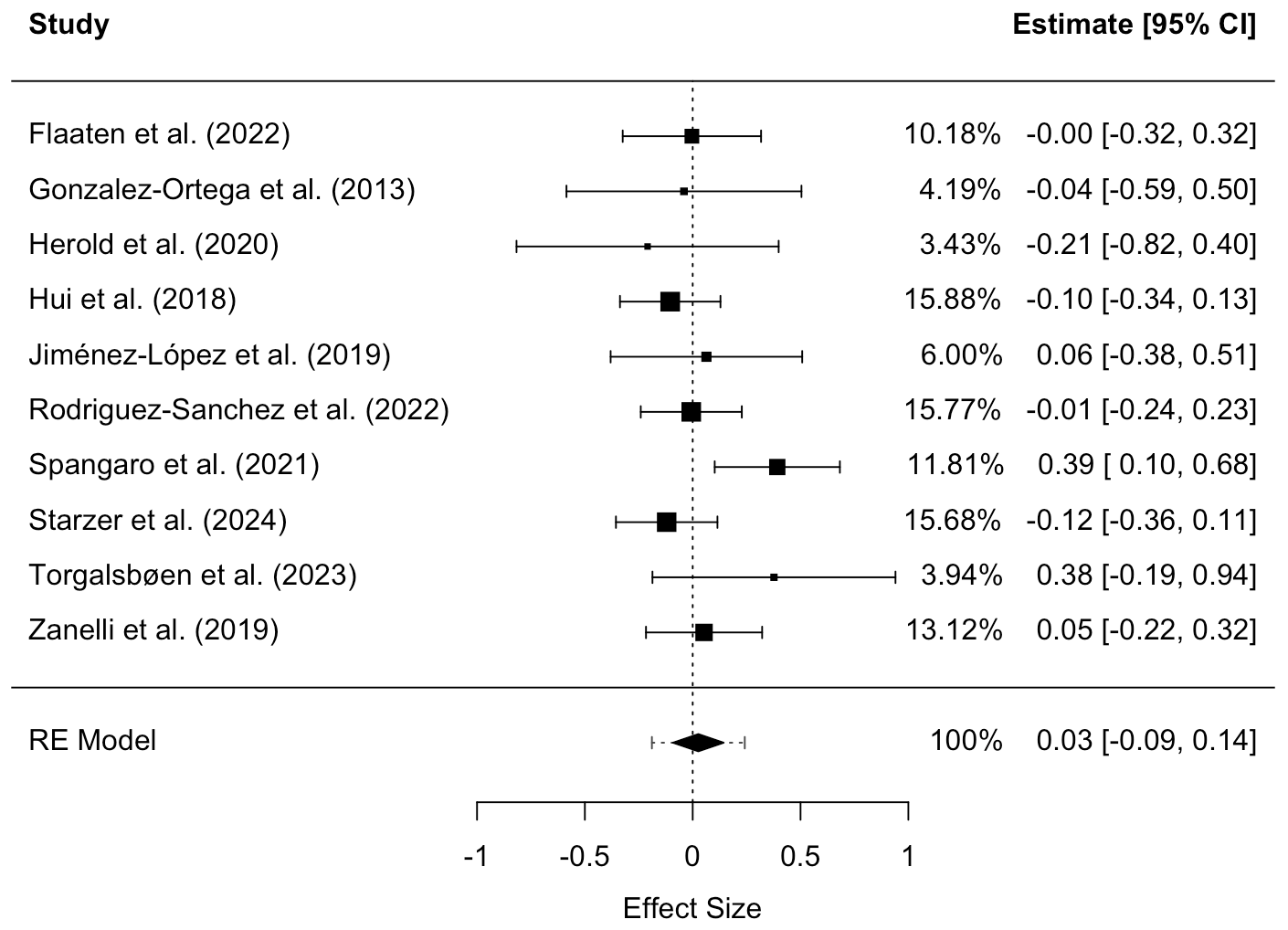


Heterogeneity: *I^2^* = 24.61%

1. Attention and Vigilance


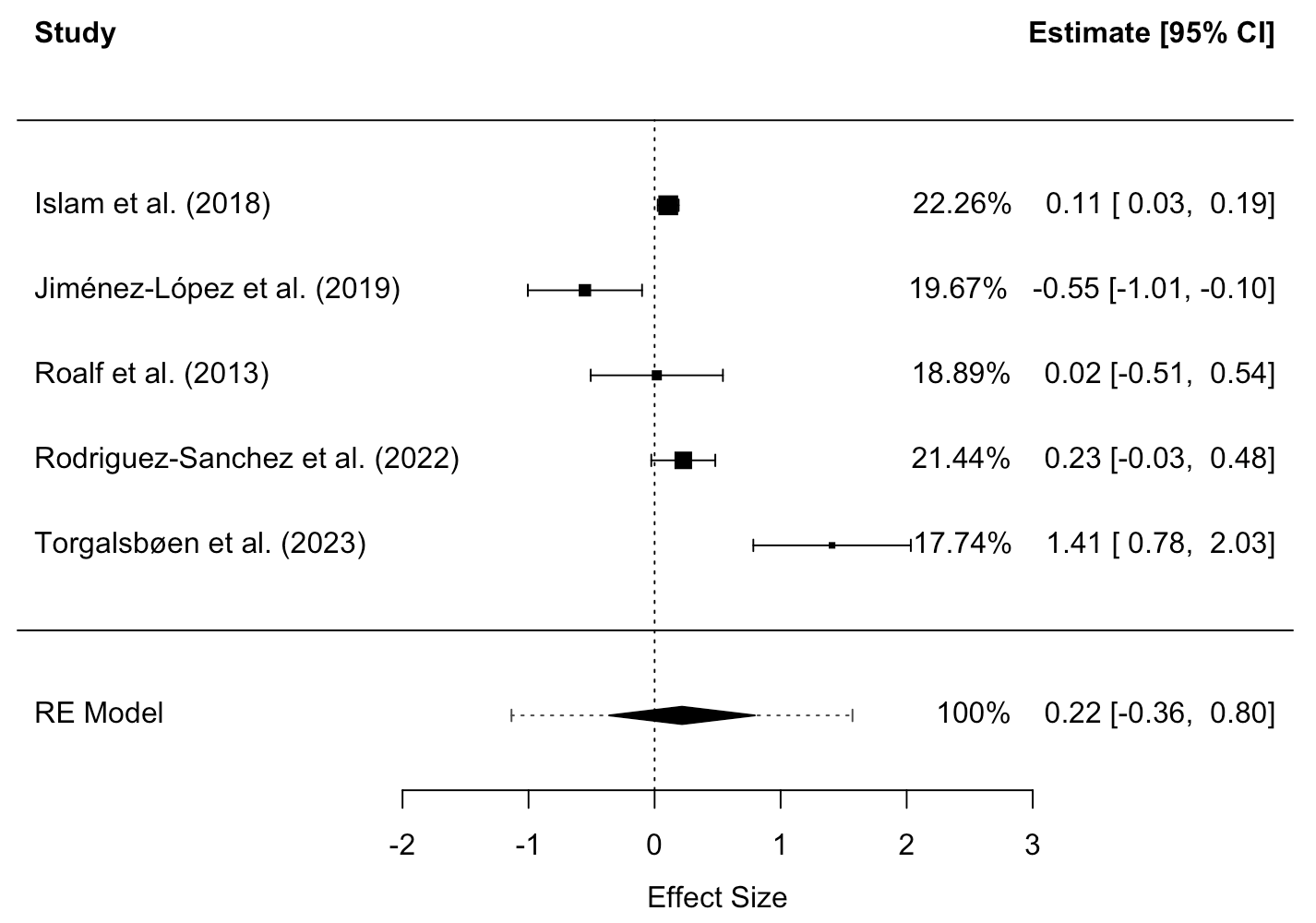


Heterogeneity: *I^2^* = 94.66%

1.
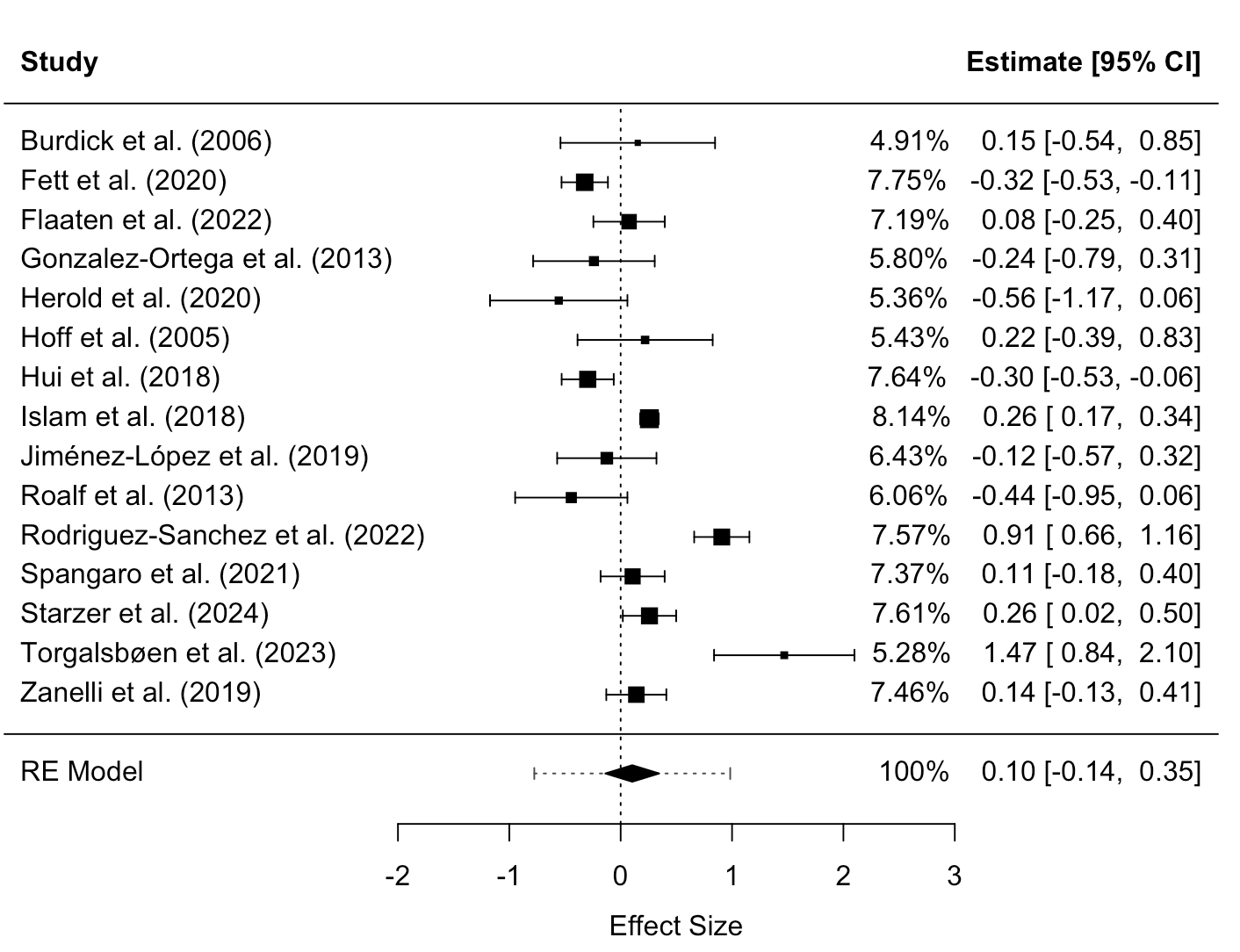
Speed of Processing

Heterogeneity: *I^2^* = 90.88%

1.
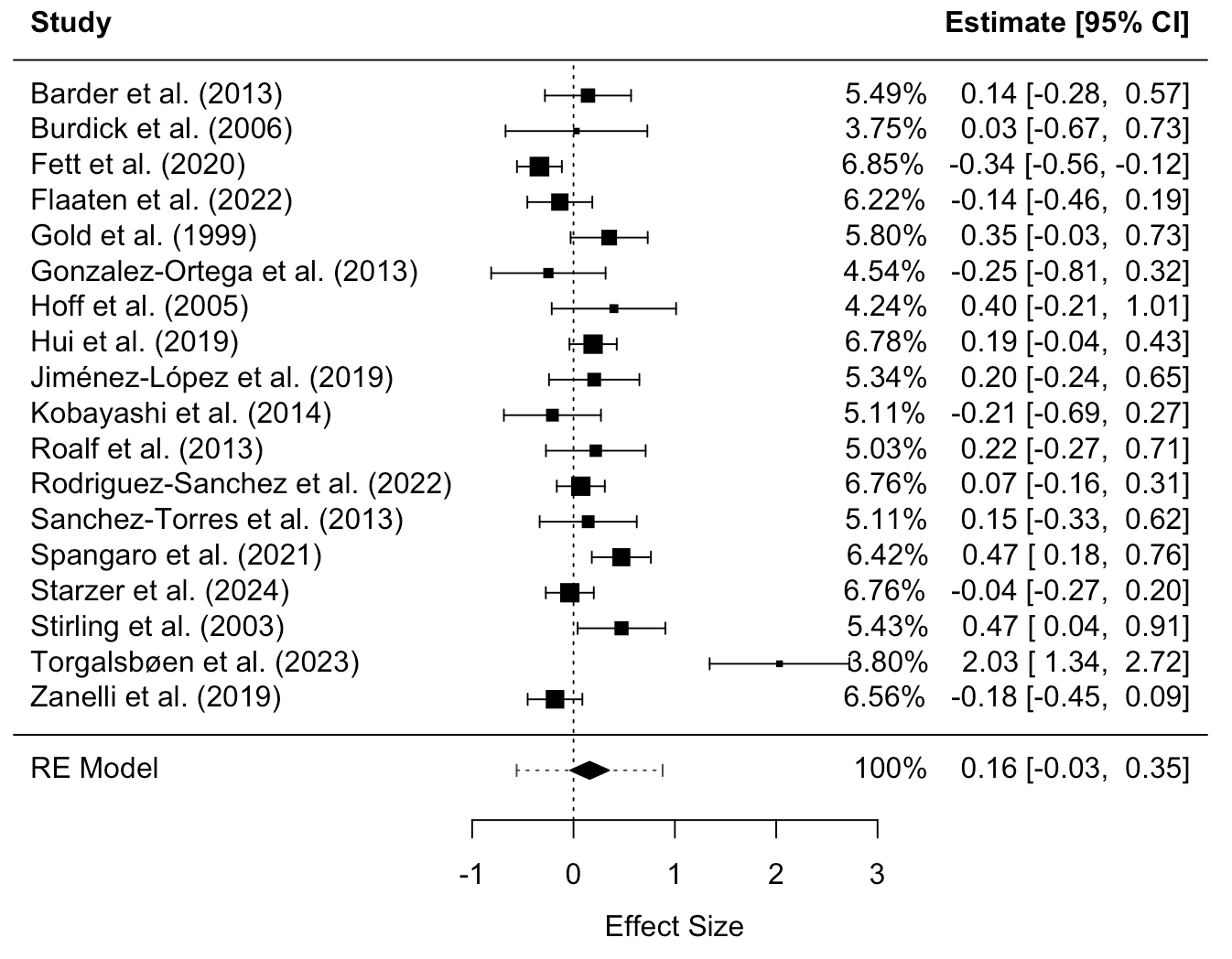
Reasoning and Problem-Solving

Heterogeneity: *I^2^* = 80.14%

1. Verbal Fluency


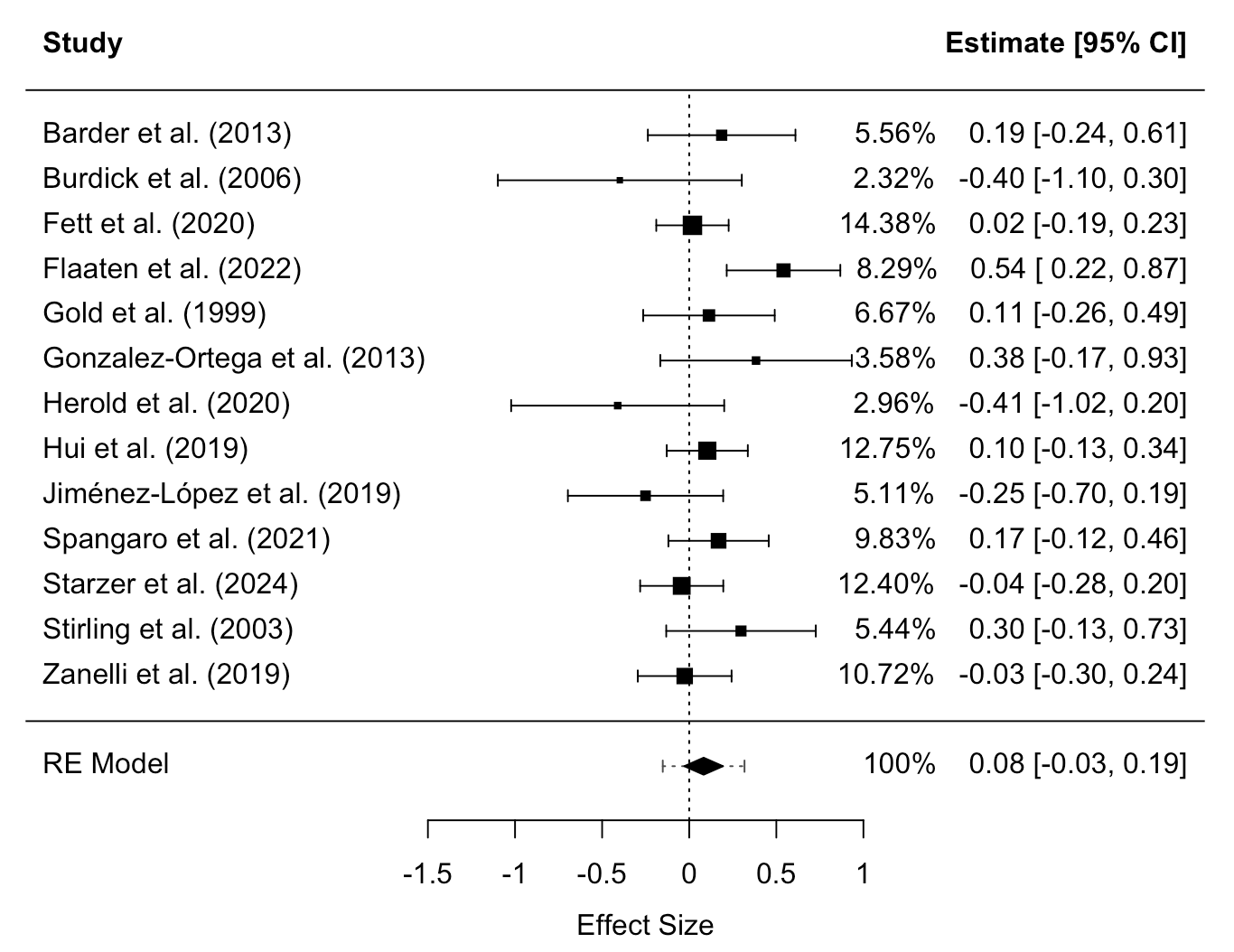


Heterogeneity: *I^2^* = 28.38%

**eFigure 3. Forest Plots for the Within-Subject Change in Controls for Each Domain.**

The estimate represents the effect size (Hedge’s g). RE model= random-effects model. The percentages represent the weights of each study.

1. Global Cognition


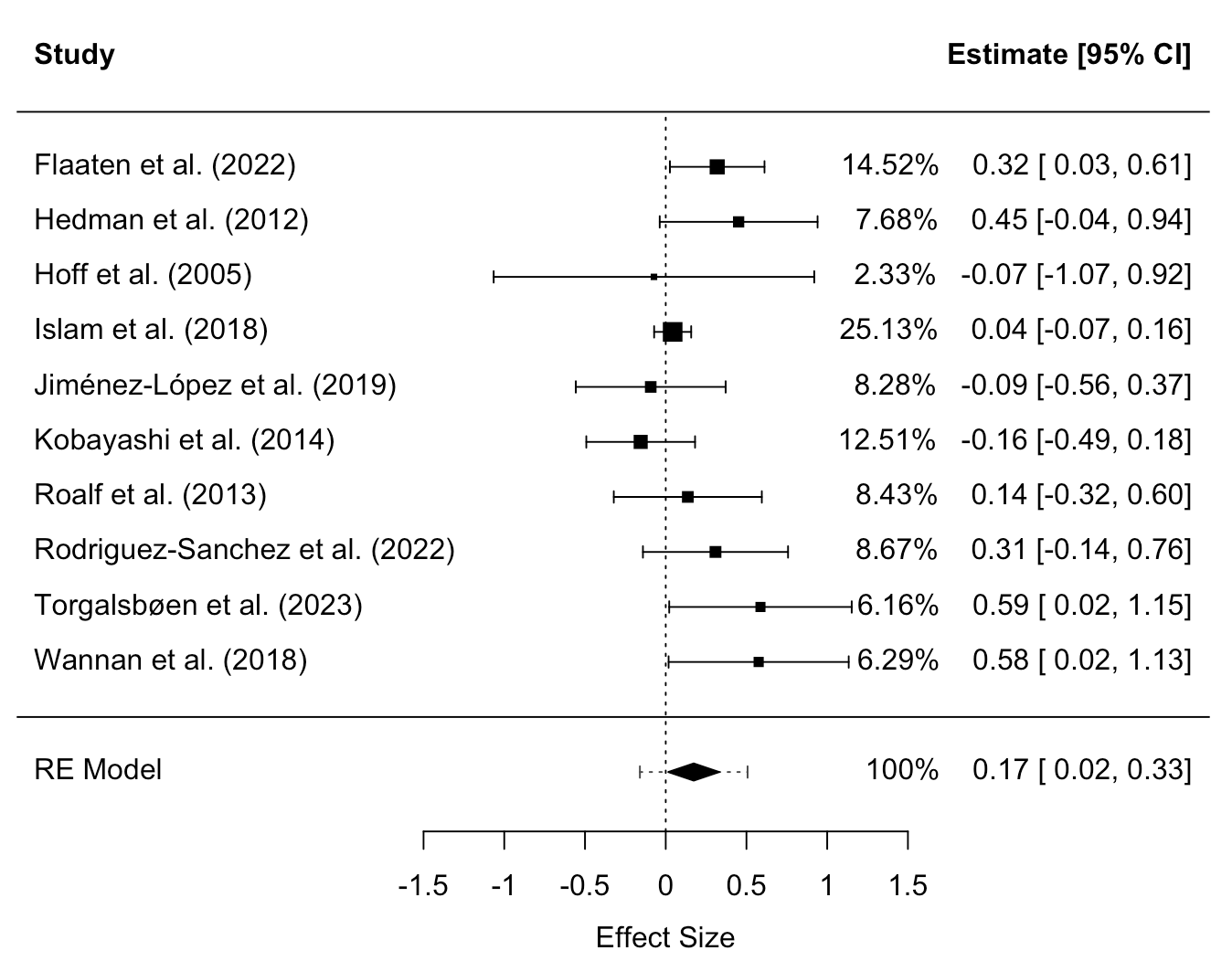


Heterogeneity: *I^2^* =41.01%

1. Verbal Learning and Memory


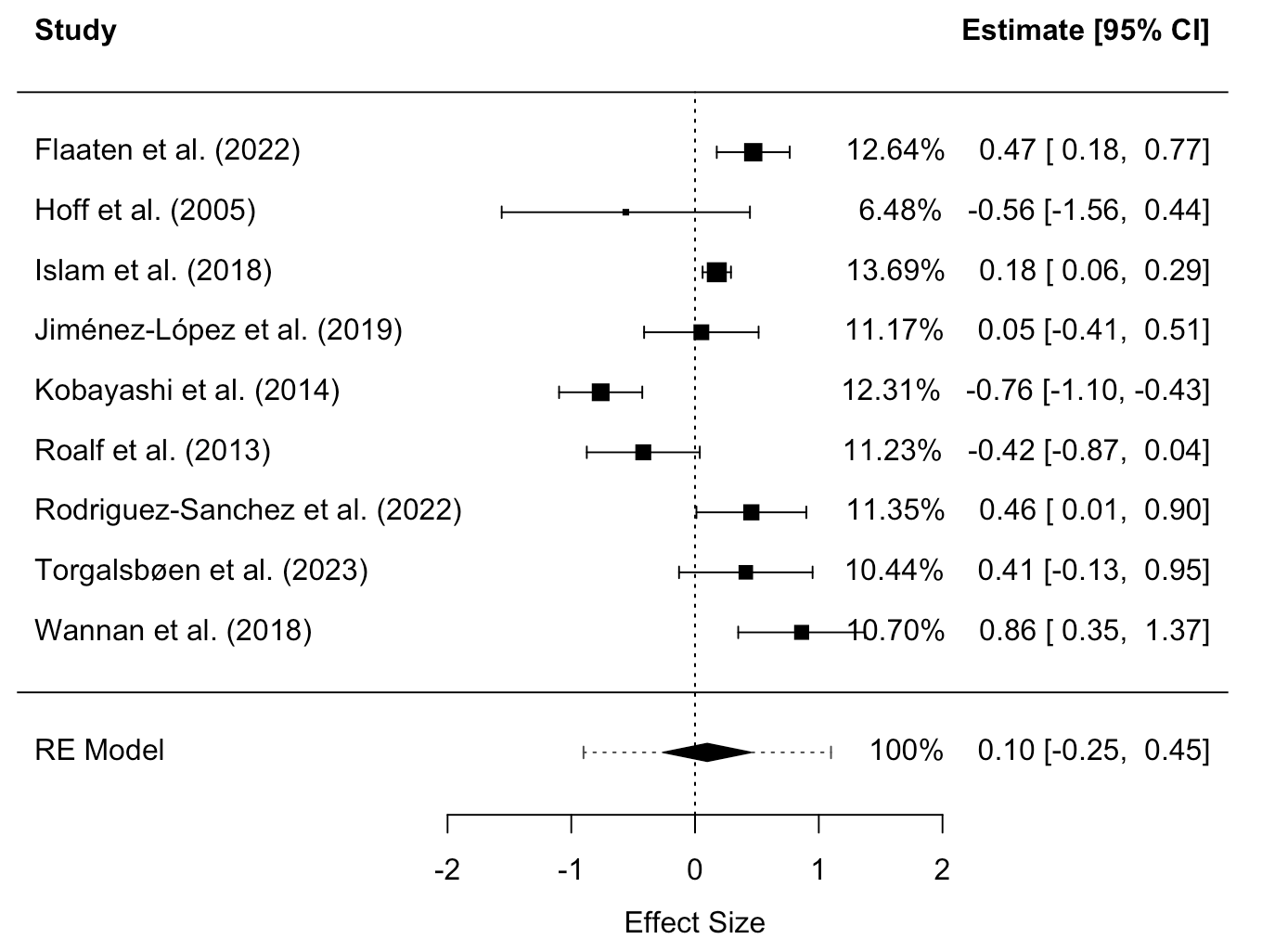


Heterogeneity: *I^2^* = 88.19%

1.
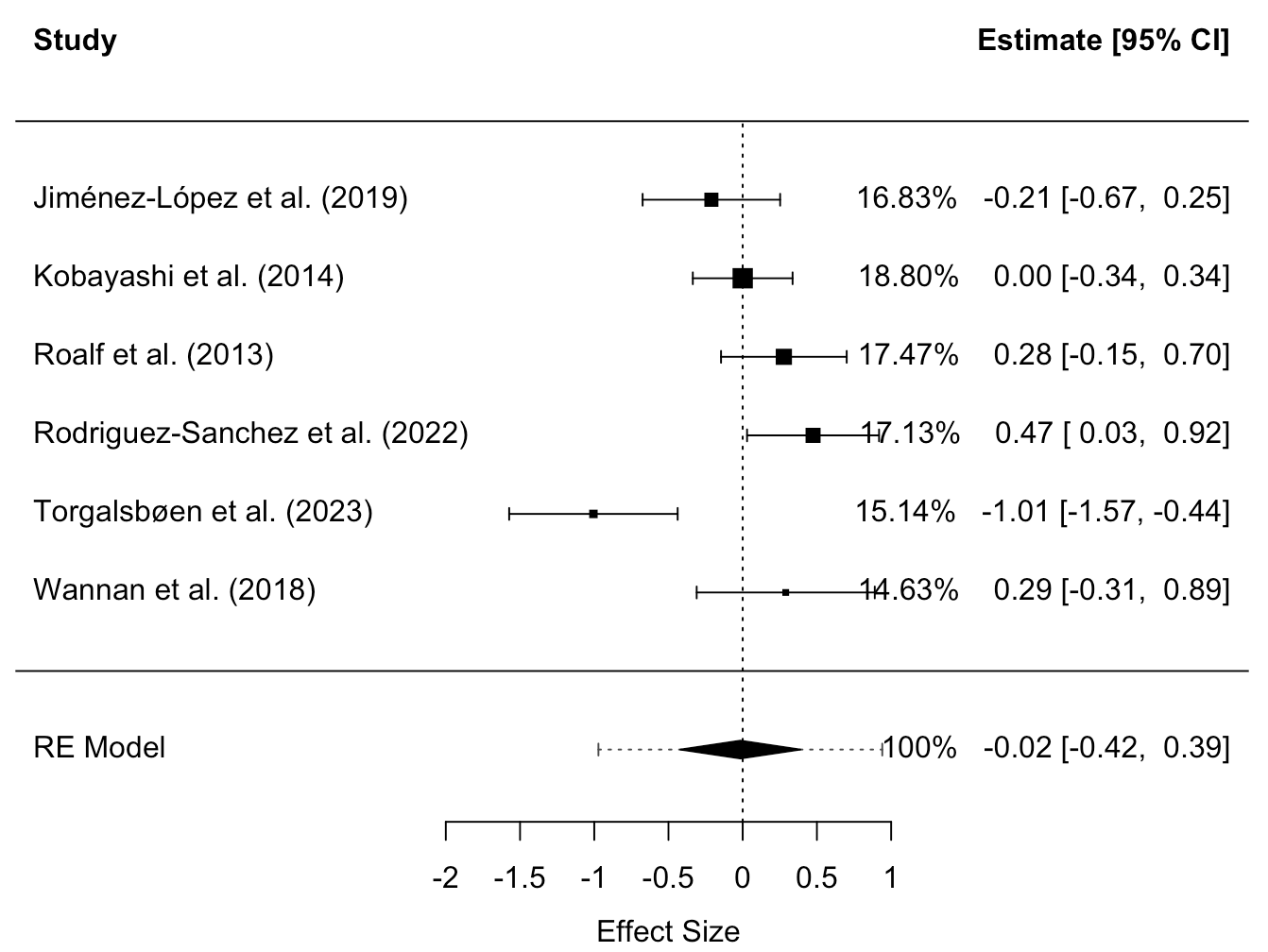
Visual Learning and Memory

Heterogeneity: *I^2^* = 78.46%

1.
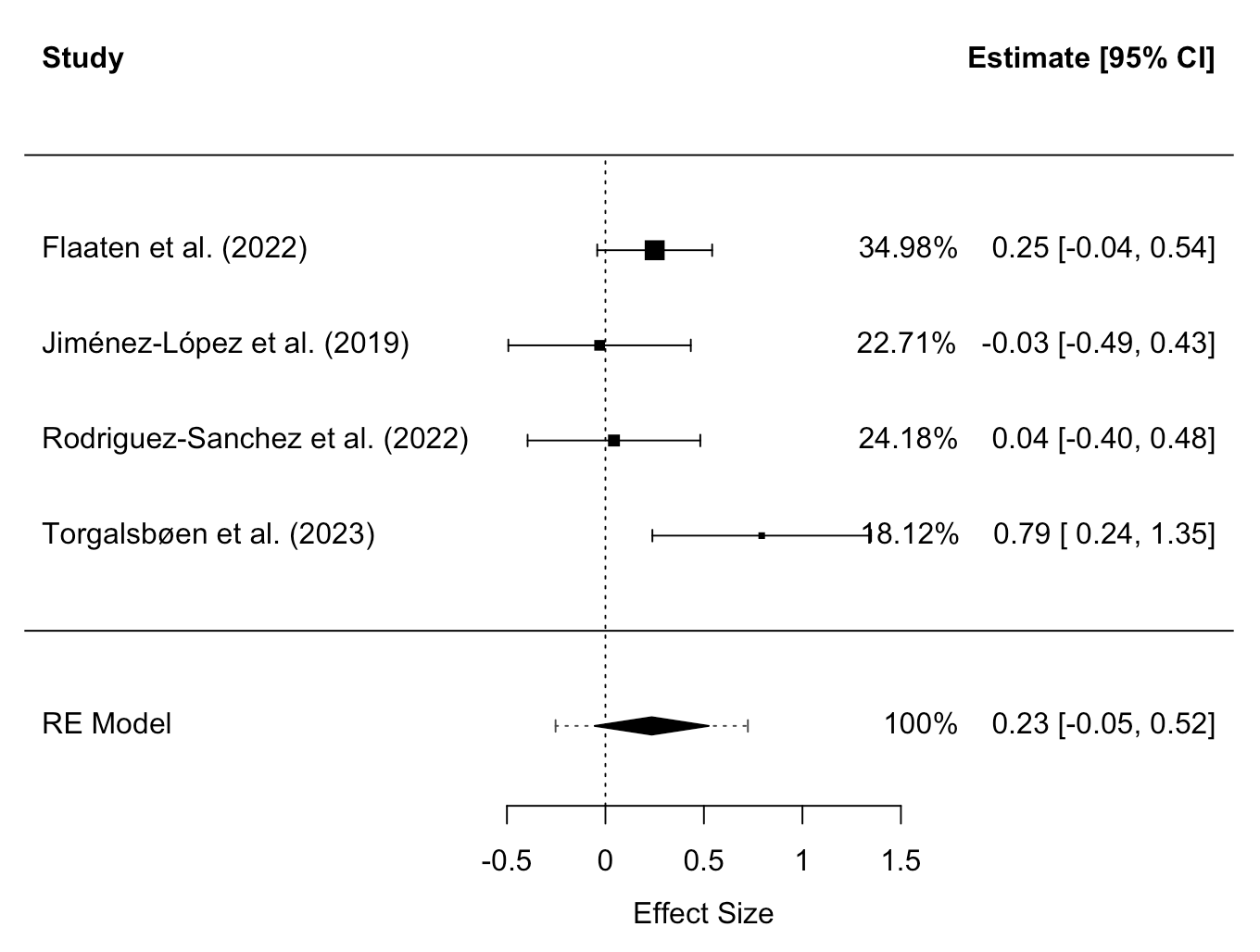
Working Memory

Heterogeneity: *I^2^* = 31.94%

1. Attention and Vigilance


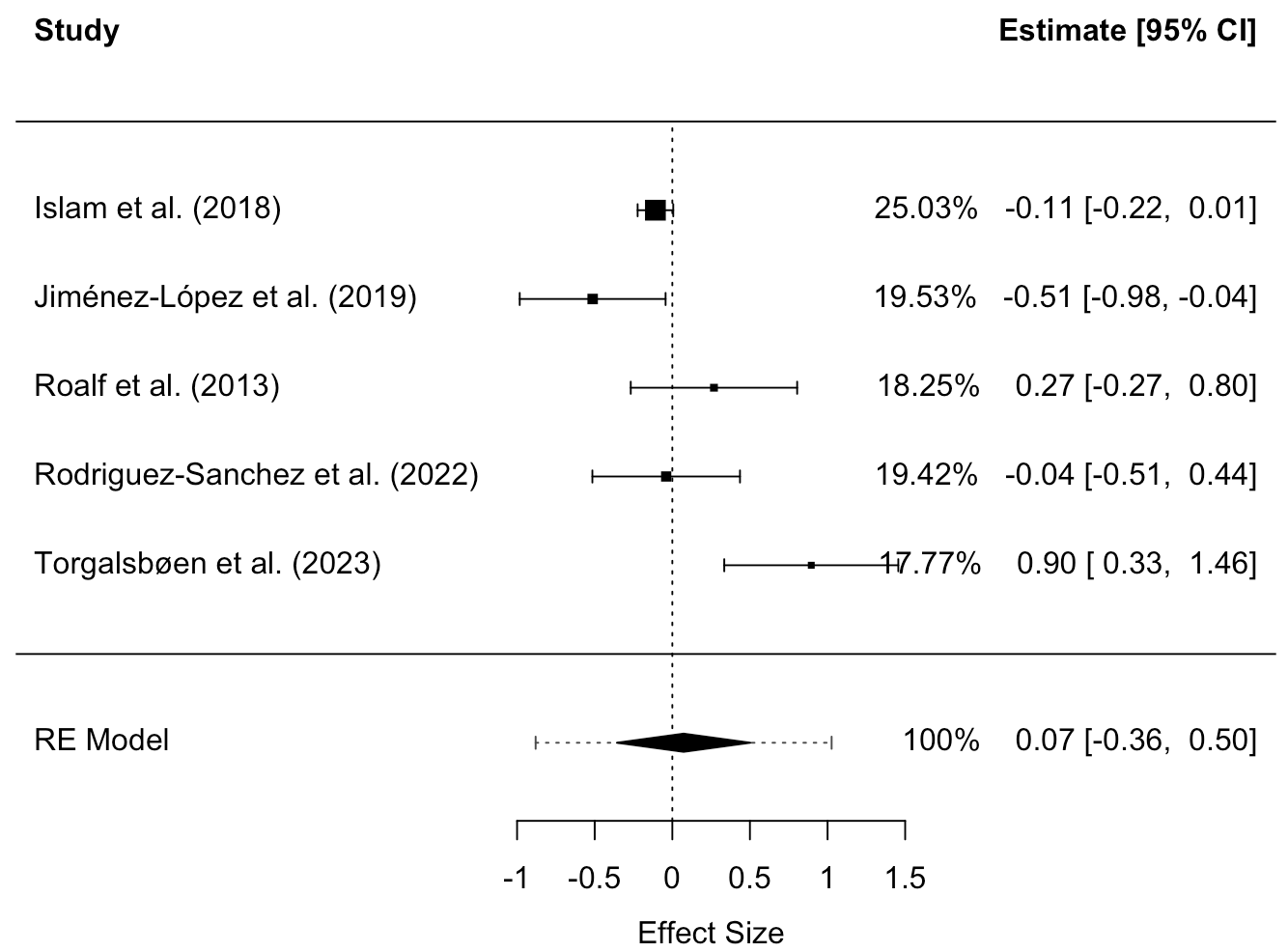


Heterogeneity: *I^2^* = 83.47%

1. Speed of Processing


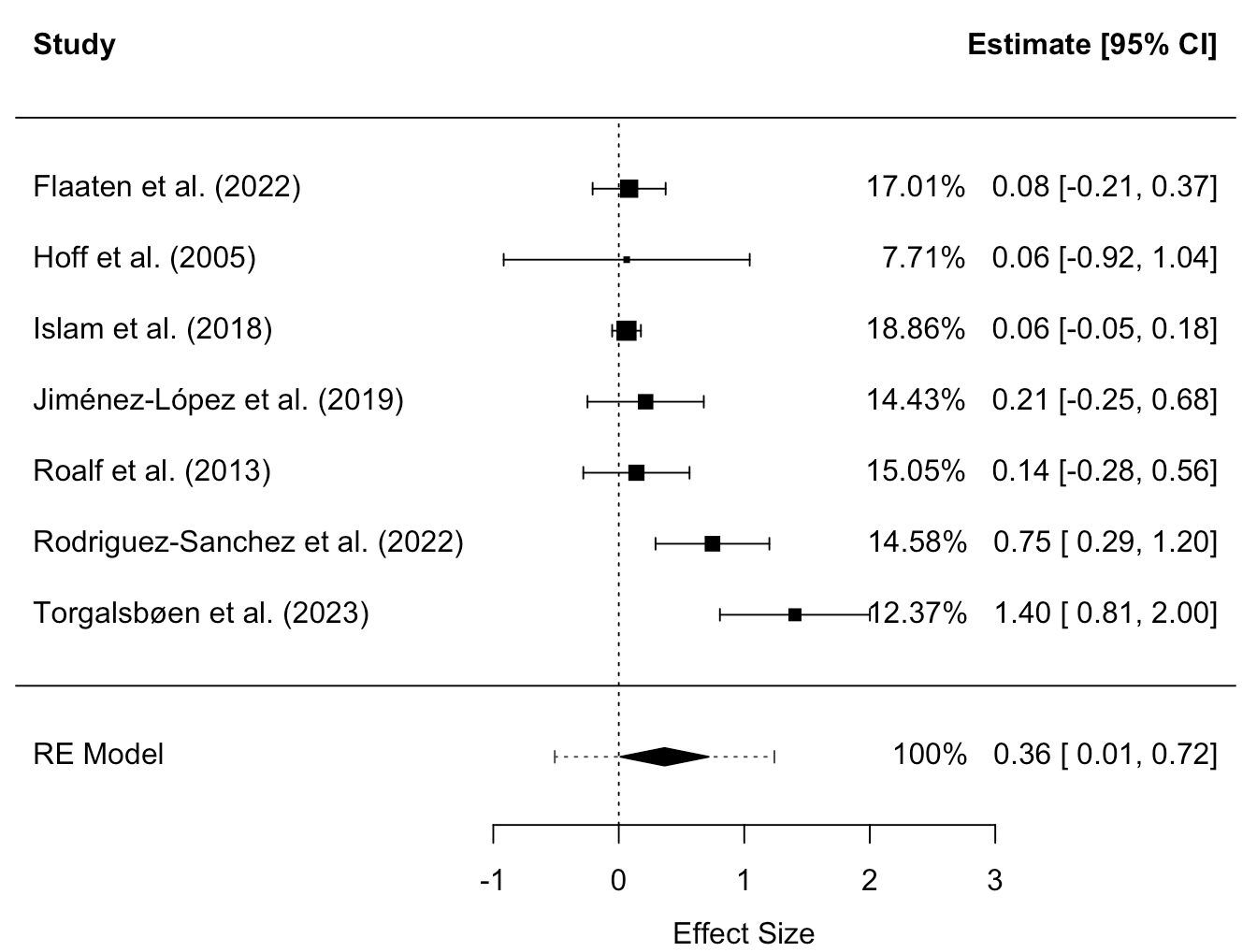


Heterogeneity: *I^2^* = 84.41%

1. Reasoning and Problem-Solving


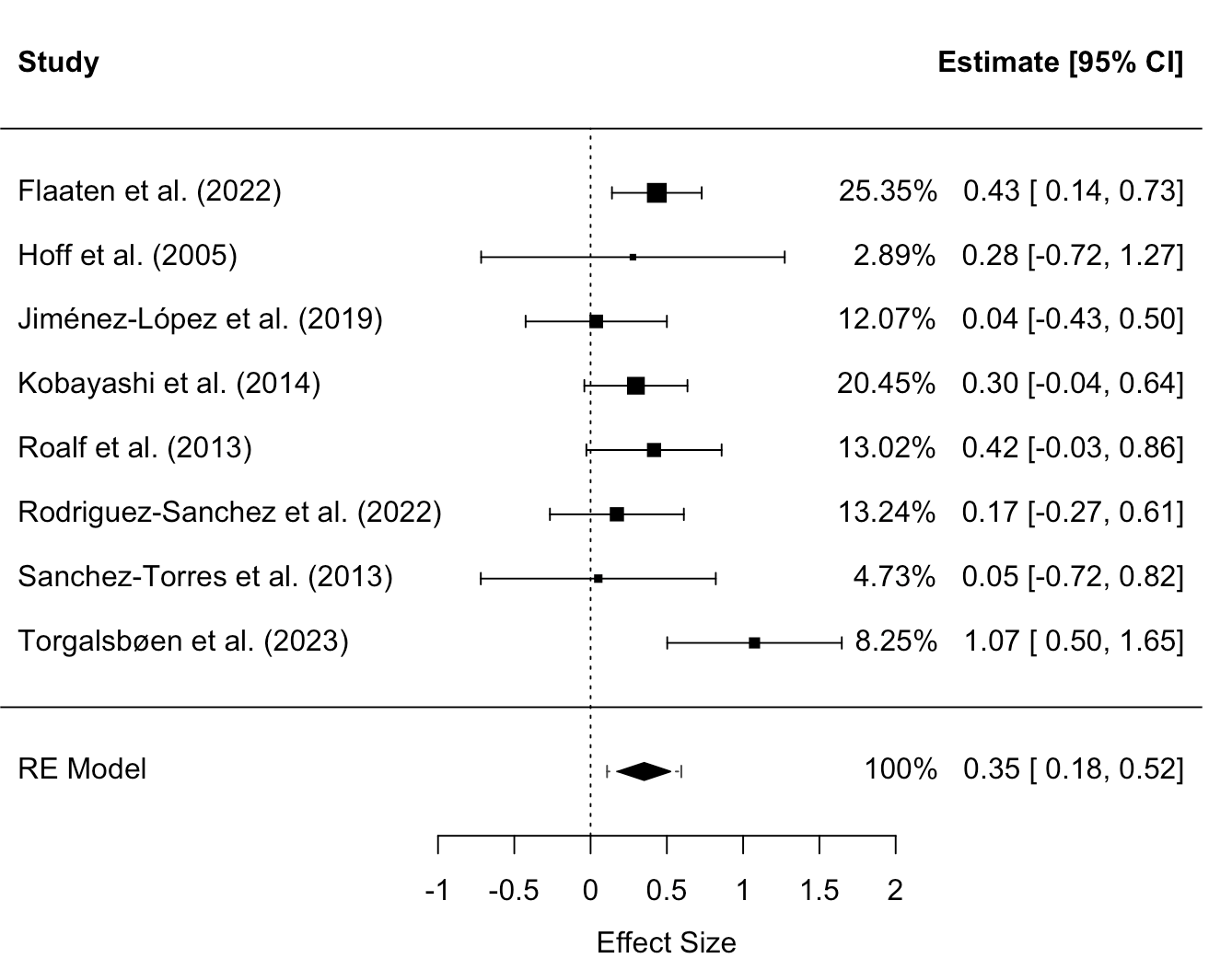


Heterogeneity: *I^2^* = 12.55%

**eFigure 4. Funnel Plots Per Domain for the Within-Subject Change Over Time in Patients**

The funnel plots presented below are for domains with a minimum of 10 studies.

1.
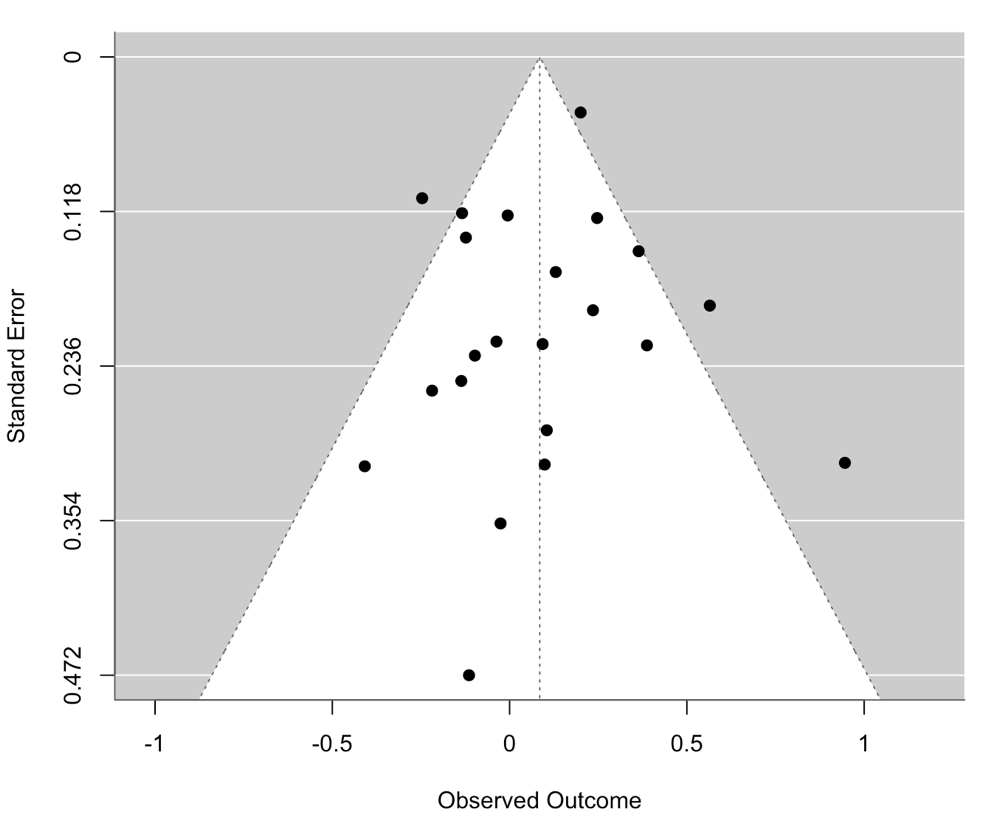
Global Cognition
2. Verbal Learning and Memory


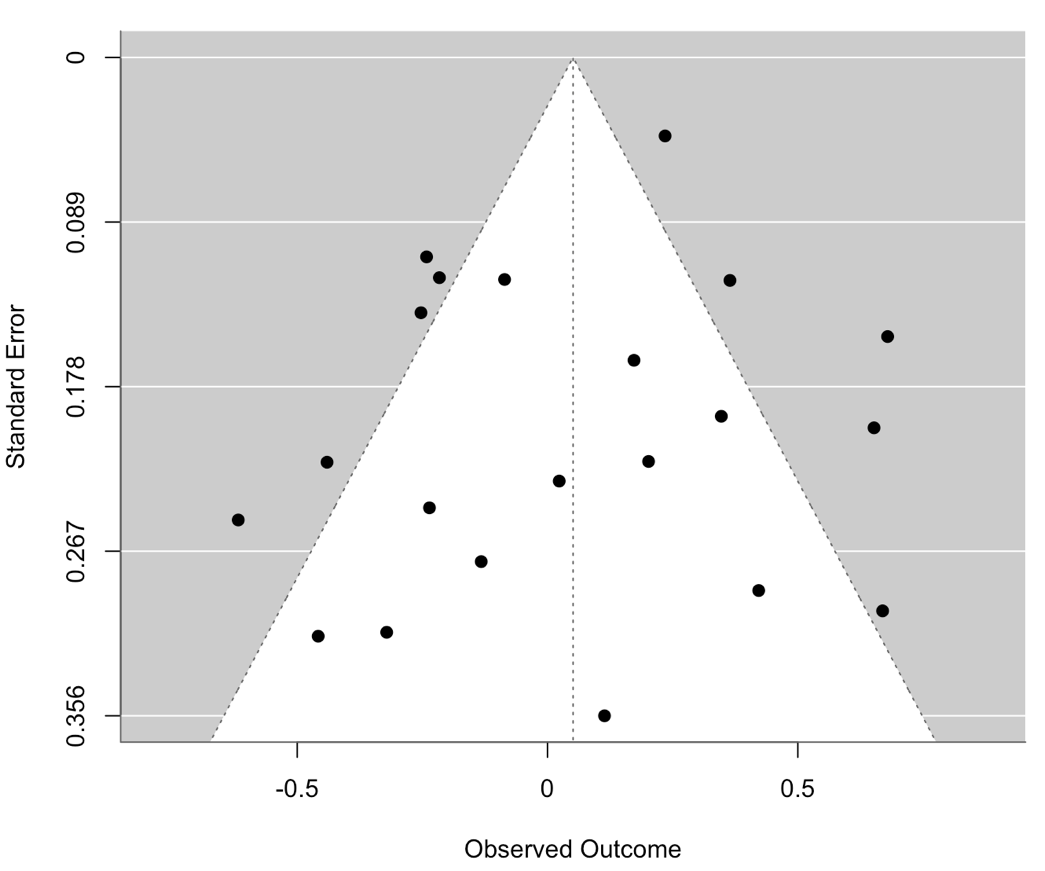


1. Visual Learning and Memory


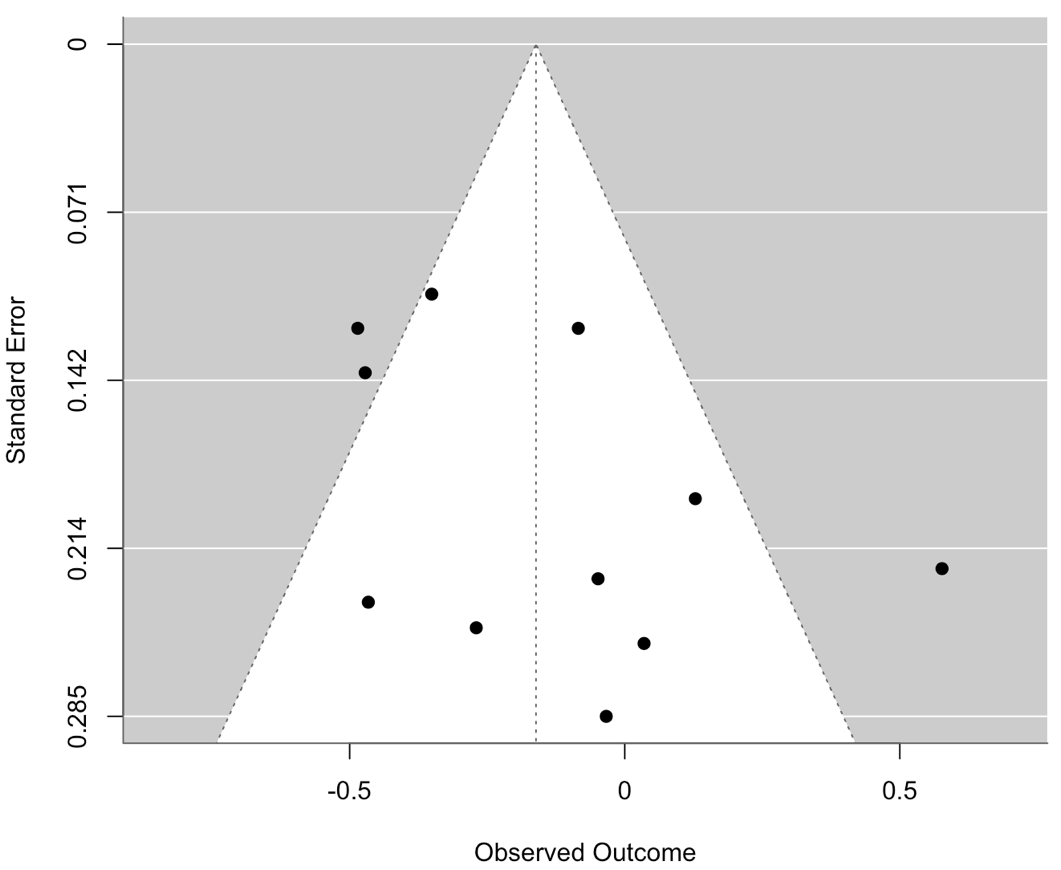


1. Working Memory


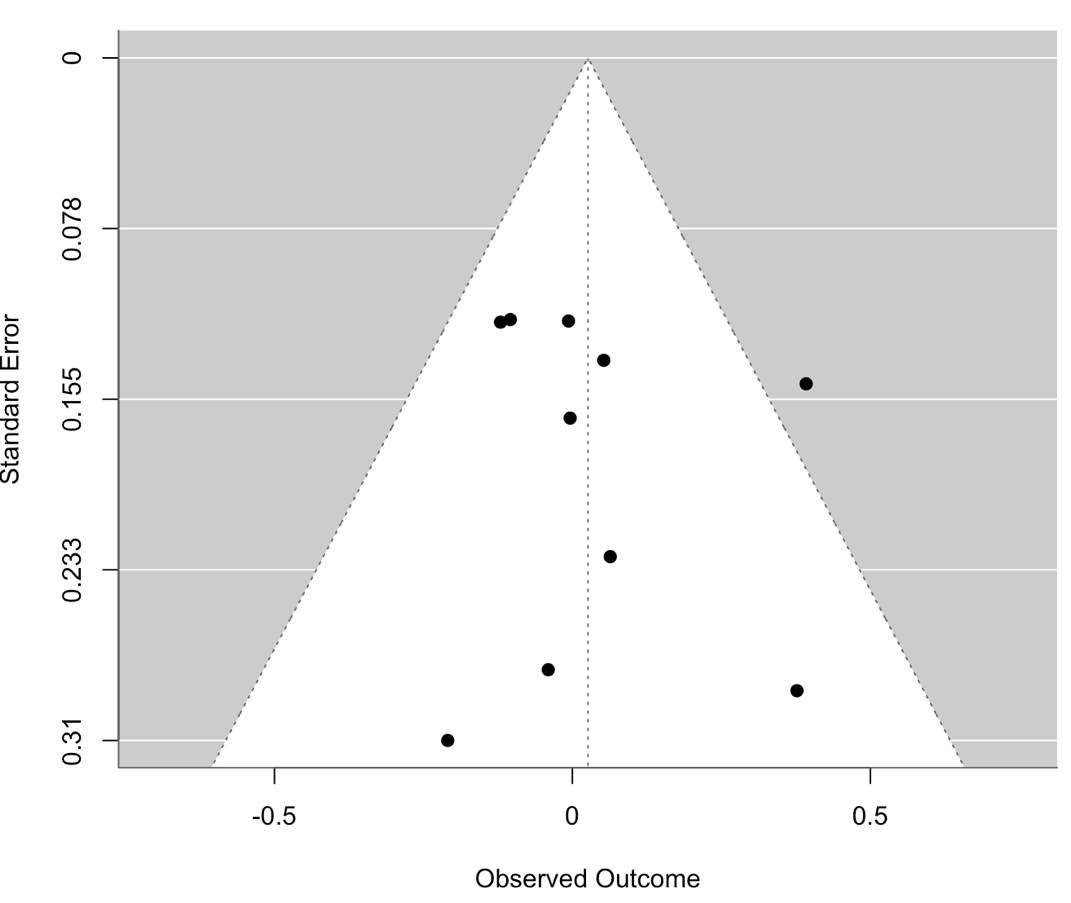


1. Speed of Processing


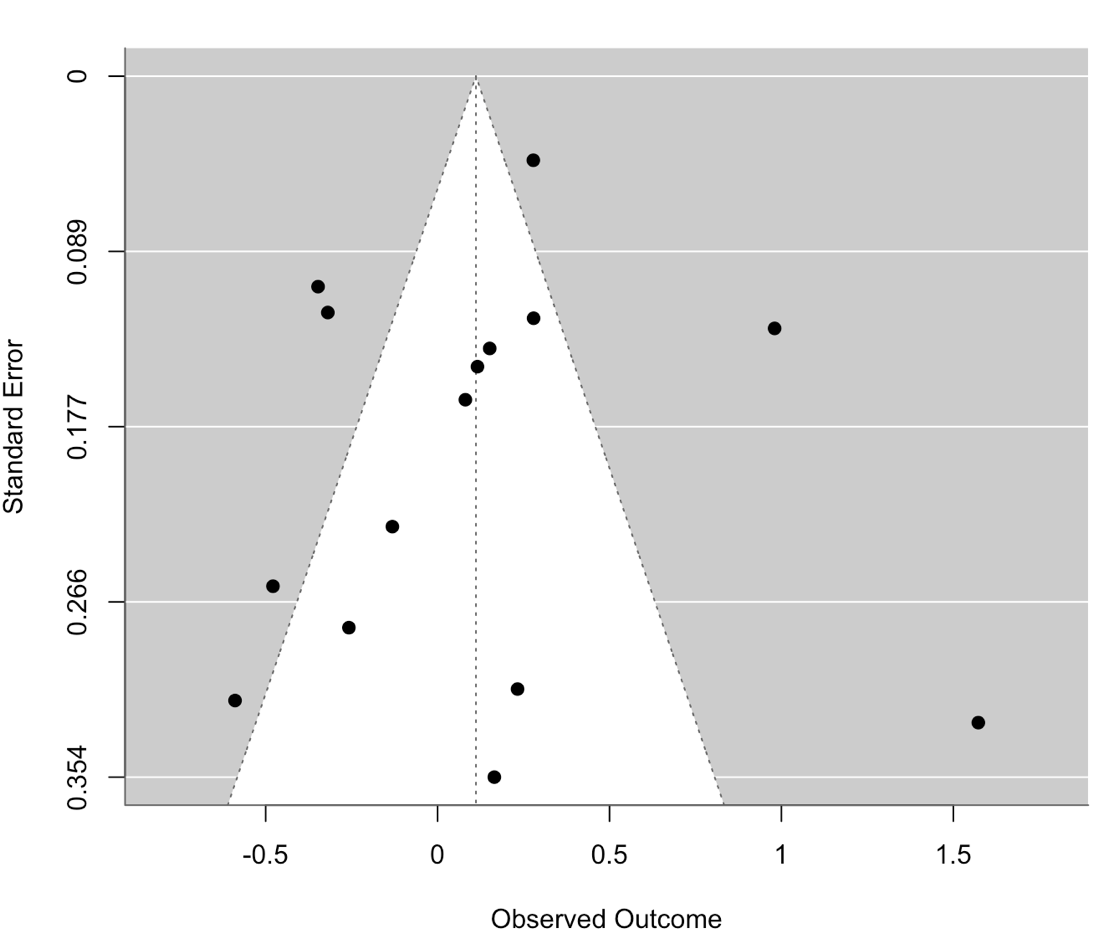


1. Reasoning and Problem-Solving


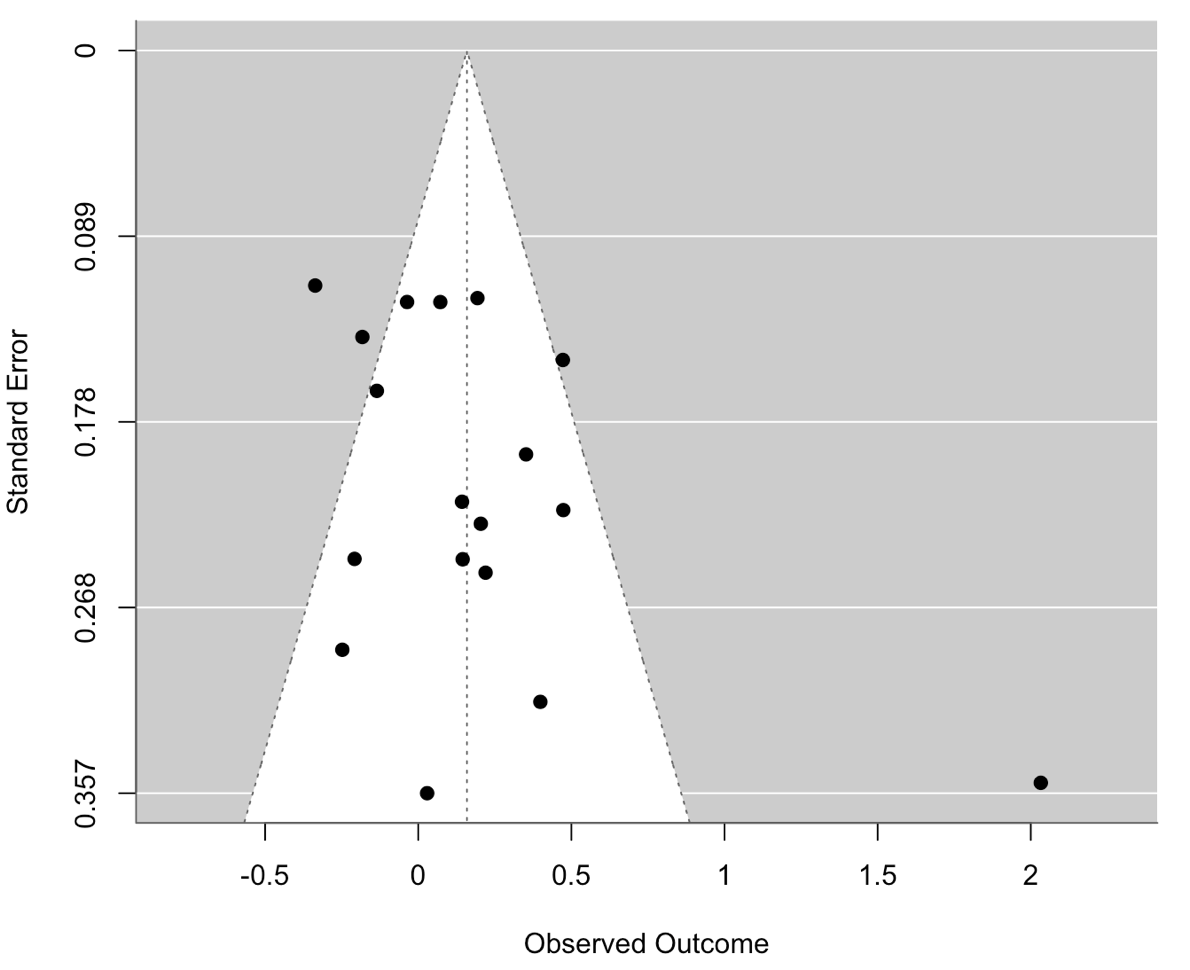


1. Verbal Fluency


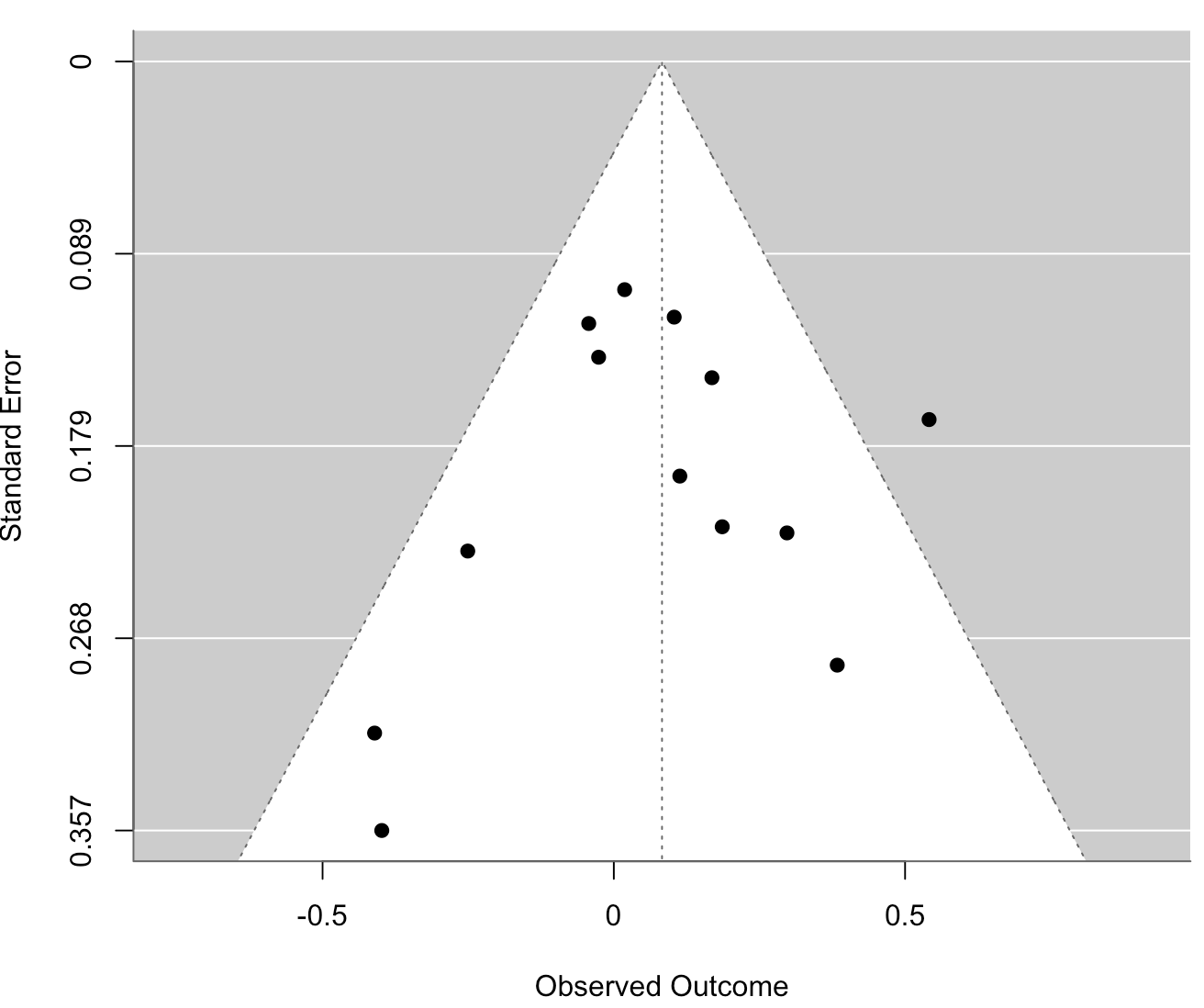


**eTable 8. Table of Outliers**

| **Domain** | **Studies (patients)** | **Studies (controls)** |
| --- | --- | --- |
| **Global Cognition** | Fett et al. (2020)  Torgalsbøen et al. (2023) | NA |
| **Verbal Learning and Memory** | Roalf et al. (2013)  Spangaro et al. (2021)  Wannan et al. (2018) | Kobayashi et al. (2014) |
| **Visual Learning and Memory** | Stirling et al. (2003) | Torgalsbøen et al. (2023) |
| **Working Memory** | NA | NA |
| **Attention and Vigilance** | NA | NA |
| **Speed of Processing** | Rodriguez-Sanchez et al. (2022)  Torgalsbøen et al. (2023) | Torgalsbøen et al. (2023) |
| **Reasoning & Problem-Solving** | Fett et al. (2020)  Torgalsbøen et al. (2023) | NA |
| **Verbal Fluency** | NA | NA |

*Note.* Studies identified as outliers using the “find.outliers” function from the “dmetar” package for patients and controls separately. NA indicates that no outliers were identified, or that between-study heterogeneity was under 50%.

**eTable 9. Main Results Excluding Outliers**

| **Neurocognitive domain** | **k** | **Hedge’s g (95% CI)** | **Z** | **P** | **Q** | **Q(p)** | **I^2^%** | **Bias(p)** | **QM(p)** |
| --- | --- | --- | --- | --- | --- | --- | --- | --- | --- |
| **Global Cognition** |  |  |  |  |  |  |  |  |  |
| Patients | 20 | . 09 (-.01; .19) | 1.83 | .07 | 30.60 | .04 | 42.68 | .12 | 8.00 (.02)* |
| Controls | 10 | .17 (.02; .33) | 2.15 | .03 | 14.55 | .10 | 41.01 | .16 |  |
| **Verbal Learning and Memory** |  |  |  |  |  |  |  |  |  |
| Patients | 18 | .01 (-.13; .15) | .15 | .88 | 61.75 | <.0001 | 70.93 | .12 | 4.13 (.13) |
| Controls | 8 | .23 (-.04; .51) | 1.67 | .10 | 21.23 | .003 | 75.72 | - |  |
| **Visual Learning and Memory** |  |  |  |  |  |  |  |  |  |
| Patients | 10 | -.24 (-.39; -.09) | -3.20 | .001 | 16.30 | .06 | 47.30 | .21 | 11.92 (.003)* |
| Controls | 5 | .15 (-.09; .38) | 1.24 | .22 | 5.68 | .22 | 30.42 | - |  |
| **Working Memory** ^a^ |  |  |  |  |  |  |  |  |  |
| Patients | 10 | .03 (-.09; .14) | .44 | .66 | 11.08 | .27 | 24.61 | .55 | 3.61 (.16) |
| Controls | 4 | .22 (-.04; .47) | 1.66 | .10 | 5.18 | .16 | 31.94 | - |  |
| **Attention and Vigilance** ^a^ |  |  |  |  |  |  |  |  |  |
| Patients | 5 | .22 (-.36; .80) | .74 | .46 | 25.70 | <.0001 | 94.66 | - | .77 (.68) |
| Controls | 5 | .07 (-.36; .50) | .33 | .74 | 16.78 | .0021 | 83.47 | - |  |
| **Speed of Processing** |  |  |  |  |  |  |  |  |  |
| Patients | 13 | -.03 (-.19; .13) | -.37 | .71 | 53.79 | <.0001 | 72.33 | .04 | 2.96 (.23) |
| Controls | 6 | .18 (-.01; .38) | 1.85 | .06 | 8.50 | .13 | 45.63 | - |  |
| **Reasoning & Problem-Solving** |  |  |  |  |  |  |  |  |  |
| Patients | 16 | .11 (-.01; .23) | 1.85 | .06 | 23.54 | .07 | 39.59 | .65 | 16.30 (.000)* |
| Controls | 8 | .35 (.18; .52) | 4.01 | <.0001 | 9.64 | .21 | 12.55 | - |  |
| **Verbal Fluency** |  |  |  |  |  |  |  |  |  |
| Patients | 12 | .04 (-.05; .13) | .87 | .38 | 10.50 | .49 | 0 | .67 | - |
| Controls ^b^ | - | - | - | - | - | - | - | - | - |

Abbreviations: k, number of studies. Q represents the Cochran’s Q value as a measure of between-study heterogeneity. *I^2^*  represents the percentage of variation between studies explained by heterogeneity (25% low heterogeneity, 50% moderate heterogeneity, 75% high heterogeneity). Bias represents the p-value of the egger’s test used to measure publication bias (reported when k=10 or more). The QM(p) is the result of the subgroup analysis comparing the change in cognition between patients and controls. *Denotes a significant difference between patients and controls (p<.05).

^a^No outliers detected using the “find.outliers” function

^b^Not enough studies assessing verbal fluency in controls (k=2).

**eTable 10. Meta-Analyses Results of the Comparison of Change Scores Over Time in Studies Including Both Patients and Controls**

| **Neurocognitive domain** | **k** | **Hedge’s g (95% CI)** | **Z** | **P** | **Q** | **Q(p)** | **I^2^%** | **Bias(p)** |
| --- | --- | --- | --- | --- | --- | --- | --- | --- |
| Global Cognition | 10 | -.06 (-.24; .11) | -.72 | .47 | 21.03 | .01 | 51.93 | .05 |
| Verbal Learning and Memory | 9 | -.05 (-.20; .09) | -.70 | .48 | 12.68 | .12 | 37.63 | - |
| Visual Learning and Memory | 6 | -.17 (-.73; .40) | -.58 | .56 | 40.60 | <.0001 | 89.60 | - |
| Working Memory | 4 | .14 (-.33; .05) | -1.40 | .16 | 2.49 | .48 | 0 | - |
| Attention and Vigilance | 5 | .21 (-.19; .62) | 1.04 | .30 | 15.43 | .004 | 84.17 | - |
| Speed of Processing | 7 | .01 (-.27; .29) | .10 | .92 | 22.08 | .001 | 77.54 | - |
| Reasoning & Problem-Solving | 8 | .01 (-.02; .32) | .06 | .95 | 25.25 | .0007 | 74.21 | - |
| Verbal Fluency^a^ | - | - | - | - | - | - | - | - |

*Note*. Effect size (Hedge’s g) of rate of change in cognitive performance for the included neurocognitive domains over time between patients and controls. k=number of studies. Q represents the Cochran’s Q value as a measure of between-study heterogeneity. *I^2^*  represents the percentage of variation between studies explained by heterogeneity (25% low heterogeneity, 50% moderate heterogeneity, 75% high heterogeneity). Bias represents the p-value of the egger’s test used to measure publication bias (reported when k=10 or more). ^a^Not enough studies assessing verbal fluency in both patients and controls (n=2).

**eTable 11. Meta-Regression Results for Age, Sex, and Diagnosis**

| **Domain and Variable** | **k** | **Estimate (95% CI)** | **SE** | **Z** | **QM(p)** |
| --- | --- | --- | --- | --- | --- |
| **Global Cognition** |  |  |  |  |  |
| Age | 17 | -.01 (-.03; .005) | .01 | -1.43 | 2.06 (.15) |
| Sex (%male) | 16 | .01 (-.0007; .017) | .005 | 1.81 | 3.28 (.07) |
| Diagnosis (% schizophrenia) | 22 | .003 (-.002; .01) | .003 | 1.13 | 1.28 (.26) |
| **Verbal Learning and Memory** |  |  |  |  |  |
| Age | 18 | -.03 (-.05; -.004) | .01 | -2.29 | 5.25 (.02)* |
| Sex (%male) | 17 | .01 (-.004; .03) | .01 | 1.44 | 2.06 (.15) |
| Diagnosis (% schizophrenia) | 21 | -.001 (- .008; .006) | .004 | -.29 | .08 (.77) |
| **Visual Learning and Memory** |  |  |  |  |  |
| Age | 9 | .01 (-.01; .04) | .01 | .85 | 0.73 (.39) |
| Sex (%male) | 9 | .01 (-.01; .03) | .01 | .99 | .97 (.32) |
| Diagnosis (% schizophrenia) | 11 | .008 (.0004; .016) | .004 | 2.10 | 4.41 (.04)* |
| **Working Memory** |  |  |  |  |  |
| Age | 8 | .00 (-.02; .02) | .01 | -.28 | .08 (.78) |
| Sex (%male) | 8 | .005 (-.01; .02) | .01 | .53 | .28 (.60) |
| Diagnosis (% schizophrenia) | 10 | .007 (.001; .013) | .003 | 2.18 | 4.73 (.03)* |
| **Attention** |  |  |  |  |  |
| Age | 4 | -.03 (-.06; .01) | .02 | -1.32 | 1.73 (.19) |
| Sex (%male) | 4 | .01 (-.02; .05) | .02 | .75 | .56 (.45) |
| Diagnosis (% schizophrenia) | 5 | .01 (-.02; .05) | .02 | .70 | .49 (.48) |
| **Speed of Processing** |  |  |  |  |  |
| Age | 13 | -.02 (-.05; .02) | .02 | -1.02 | 1.04 (.31) |
| Sex (%male) | 12 | .002 (-.02; .03) | .01 | .16 | .03 (.87) |
| Diagnosis (% schizophrenia) | 15 | .001 (-.01; .01) | .007 | .17 | .03 (.87) |
| **Reasoning & Problem-Solving** |  |  |  |  |  |
| Age | 15 | .00 (-.02; .02) | .01 | .10 | .01 (.92) |
| Sex (%male) | 14 | .01 (-.01; .02) | .01 | .66 | .43 (.51) |
| Diagnosis (% schizophrenia) | 18 | .005 (-.004; .014) | .005 | .99 | .98 (.32) |
| **Verbal Fluency** |  |  |  |  |  |
| Age | 11 | -.03 (-.05; -.008) | .01 | -2.76 | 7.61 (.01)* |
| Sex (%male) | 10 | -.002 (-.02; .01) | .01 | -.24 | .06 (.81) |
| Diagnosis (% schizophrenia) | 13 | -.0004 (-.006; .006) | .003 | -.13 | .02 (.90) |

Abbreviations: k,number of studies, SE, standard error, Z, z-statistic value. The QM(p) are the test statistic and p-value of the results of the meta-regression analyses. *Denotes a significant moderator for the given domain (p<.05).

**eFigure 4. Scatterplots of the Percent Schizophrenia Meta-Regression for Visual Learning and Memory and Working Memory**

Visual learning and Memory


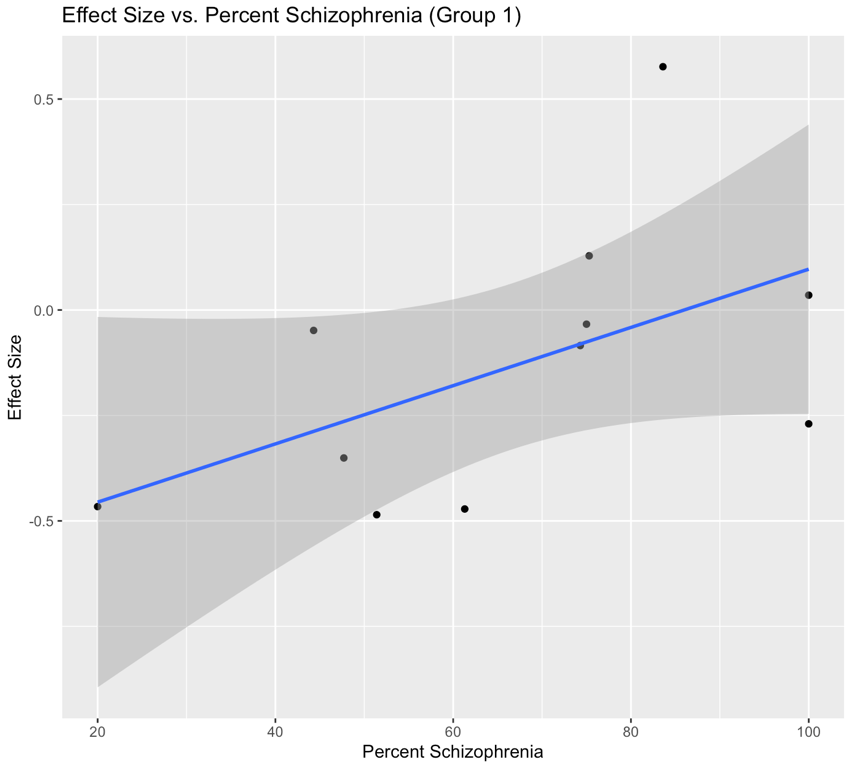


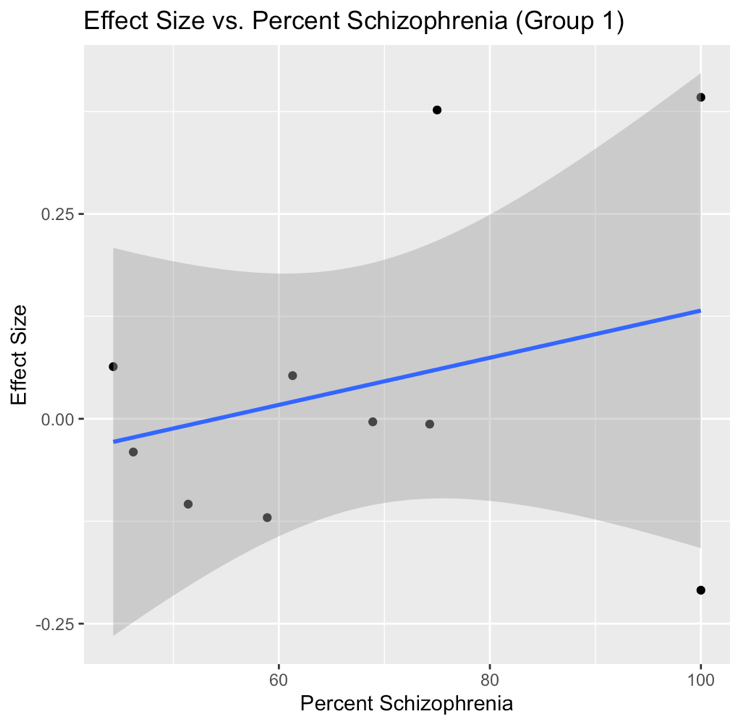
 Working Memory

**eTable 12. Results of the Follow-Up Period Subgroup Analysis**

| **Domain** | **k** | **Hedge’s g (95% CI)** | **SE** | **Z** | **QM(p)** |
| --- | --- | --- | --- | --- | --- |
| **Global Cognition** |  |  |  |  |  |
| 5-9 years | 11 | .06 (-.11; .24) | .09 | .70 | 2.14 (.34) |
| 10+ years | 11 | .10 (-.05; .26) | .08 | 1.28 |  |
| **Verbal Learning and Memory** |  |  |  |  |  |
| 5-9 years | 11 | .15 (-.07; .38) | .11 | 1.34 | 1.95 (.38) |
| 10+ years | 10 | -.04 (-.26; .17) | .11 | -.40 |  |
| **Visual Learning and Memory** |  |  |  |  |  |
| 5-9 years | 5 | -.12 (-.43; .20) | .16 | -.73 | 2.66 (.26) |
| 10+ years | 6 | -.19 (-.44; .06) | .13 | -1.46 |  |
| **Working Memory** |  |  |  |  |  |
| 5-9 years | 4 | .19 (-.02; .39) | .11 | 1.75 | 3.31 (.19) |
| 10+ years | 6 | -.03 (-.14; .08) | .06 | -.51 |  |
| **Speed of Processing** |  |  |  |  |  |
| 5-9 years | 7 | -.10 (-.46; .27) | .18 | -.52 | 2.81 (.25) |
| 10+ years | 8 | .26 (-.06; .58) | .16 | 1.59 |  |
| **Reasoning & Problem-Solving** |  |  |  |  |  |
| 5-9 years | 7 | .14 (-.19; .47) | .17 | .83 | 2.54 (.28) |
| 10+ years | 11 | .17 (-.08; .43) | .13 | 1.36 |  |
| **Verbal Fluency** |  |  |  |  |  |
| 5-9 years | 6 | .004 (-.21; -.22) | .11 | .04 | 2.52 (.28) |
| 10+ years | 7 | .12 (-.03; .27) | .08 | 1.59 |  |

Abbreviations: k, number of studies, SE, standard error, Z, z-statistic value. The QM(p) are the test statistic and p-value of the results of the subgroup analyses. Attention and vigilance excluded due to k<10.

**eTable 13. Results of the Subgroup Analysis Comparing FEP studies to Other Studies**

| **Domain** | **k** | **Hedge’s g [95% CI]** | **SE** | **Z** | **QM(p)** |
| --- | --- | --- | --- | --- | --- |
| **Global Cognition** |  |  |  |  |  |
| FEP | 13 | .13 (-.03, .27) | .08 | 1.61 | 2.66 (.27) |
| SSD & Enduring | 9 | .02 (-.17, .21) | .10 | .24 |  |
| **Verbal Learning and Memory** |  |  |  |  |  |
| FEP | 12 | .10 (-.12, .31) | .11 | .86 | .75 (.69) |
| SSD & Enduring | 9 | -.01 (-.27, .25) | .13 | -.09 |  |
| **Visual Learning and Memory** |  |  |  |  |  |
| FEP | 8 | -.17 (-.40, .05) | .11 | -1.57 | 2.66 (.27) |
| SSD & Enduring | 3 | -.09 (-.51, .32) | .21 | -.45 |  |
| **Working Memory** |  |  |  |  |  |
| FEP | 6 | .01 (-.15, .16) | .08 | .07 | .41 (.81) |
| SSD & Enduring | 4 | .07 (-.14, .27) | .10 | .64 |  |
| **Speed of Processing** |  |  |  |  |  |
| FEP | 8 | .21 (-.13, .55) | .17 | 1.21 | 1.49 (.48) |
| SSD & Enduring | 7 | -.02 (-.39, .35) | .19 | -.11 |  |
| **Reasoning & Problem-Solving** |  |  |  |  |  |
| FEP | 11 | .18 (-.07, .44) | .13 | 1.42 | 2.58 (.27) |
| SSD & Enduring | 7 | .13 (-20, .45) | .17 | .76 |  |
| **Verbal Fluency** |  |  |  |  |  |
| FEP | 8 | .16 (.02, .30)^a^ | .07 | 2.23 | 5.48 (.06) |
| SSD & Enduring | 5 | -.07 (-.28, .13) | .10 | -.73 |  |

Abbreviations: k, number of studies, SE, standard error, Z, z-statistic value. The QM(p) are the test statistic and p-value of the results of the subgroup analyses. FEP, First Episode Psychosis, SSD, Schizophrenia Spectrum Disorders. Attention and Vigilance excluded due to k<10.

^a^ Statistically significant change or difference between groups.

**eTable 14. Results of the Subgroup Analysis Comparing Single Test vs. Multiple Test Studies**

| **Domain** | **k** | **Hedge’s g [95% CI]** | **SE** | **Z** | **QM(p)** |
| --- | --- | --- | --- | --- | --- |
| **Verbal Learning and Memory** |  |  |  |  |  |
| Single | 17 | .07 (-.11, .25) | .09 | .71 | .52 (.77) |
| Multiple | 4 | -.02 (-.43, .38) | .21 | -.11 |  |
| **Working Memory** |  |  |  |  |  |
| Single | 5 | .04 (-.12, .20) | .08 | .54 | .29 (.86) |
| Multiple | 5 | .004 (-.19, .20) | .10 | .04 |  |
| **Speed of Processing** |  |  |  |  |  |
| Single | 8 | .15 (-.18, .49) | .17 | .89 | .86 (.65) |
| Multiple | 7 | .04 (-.32, .41) | .19 | .24 |  |
| **Reasoning & Problem-Solving** |  |  |  |  |  |
| Single | 8 | .26 (-.02, .55) | .15 | 1.81 | 3.55 (.17) |
| Multiple | 10 | .07 (-20, .34) | .14 | .50 |  |

Abbreviations: k, number of studies, SE, standard error, Z, z-statistic value. The QM(p) are the test statistic and p-value of the results of the subgroup analyses. Attention and Vigilance due to k<10. Global cognition excluded due to only two studies having a single test. Visual learning and memory excluded due to only 1 study having used multiple tests. Verbal fluency excluded due to only having 2 studies using multiple tests.

^a^ Statistically significant change or difference between groups.

**eTable 15. Meta-Analyses Results of FEP Studies with Cognition Assessed at Baseline**

| **Neurocognitive domain** | **k** | **Hedge’s g (95% CI)** | **Z** | **P** | **Q** | **Q(p)** | **I^2^%** | **Bias(p)** | **QM(p)** |
| --- | --- | --- | --- | --- | --- | --- | --- | --- | --- |
| **Global Cognition** |  |  |  |  |  |  |  |  |  |
| Patients | 12 | .17 (.01; .32) | 2.13 | .03 | 23.67 | .01 | 53 | .12 | 12.89 (.002)* |
| Controls | 5 | .37 (.17; .57) | 3.55 | .0004 | 2.04 | .73 | 0 |  |  |
| **Verbal Learning and Memory** |  |  |  |  |  |  |  |  |  |
| Patients | 11 | .13 (-.09; .36) | 1.17 | .24 | 40.87 | <.0001 | 76 | .48 | 7.72 (.02)* |
| Controls | 5 | .48 (.28; .68) | 4.67 | <.0001 | 6.33 | .18 | 0 | - |  |
| **Visual Learning and Memory** |  |  |  |  |  |  |  |  |  |
| Patients | 7 | -.14 (-.43; .16) | -.91 | .36 | 28.63 | <.0001 | 81 | - | .52 (.77) |
| Controls | 3 | -.07 (-.98; .84) | -.16 | .88 | 17.33 | .0002 | 88.5 | - |  |
| **Working Memory** |  |  |  |  |  |  |  |  |  |
| Patients | 6 | -.004 (-.13; .12) | -.07 | .94 | 2.65 | .75 | 0 | - | 5.47 (.06) |
| Controls | 3 | .29 (-.04; .62) | 1.74 | .08 | 3.93 | .14 | 47.5 | - |  |
| **Attention and Vigilance** ^b^ |  |  |  |  |  |  |  |  |  |
| Patients | - | - | - | - | - | - | - | - | - |
| Controls | - | - | - | - | - | - | - | - |  |
| **Speed of Processing** |  |  |  |  |  |  |  |  |  |
| Patients | 7 | .31 (-.15; .76) | 1.33 | .18 | 67.17 | <.0001 | 91.7 | - | 5.14 (.08) |
| Controls | 4 | .59 (-.03; 1.20) | 1.86 | .06 | 18.07 | .0004 | 83 | - |  |
| **Reasoning & Problem-Solving** |  |  |  |  |  |  |  |  |  |
| Patients | 10 | .27 (-.08; .61) | 1.53 | .13 | 44 | <.0001 | 89 | .12 | 5.96 (.05)* |
| Controls | 4 | .49 (.10; .87) | 2.49 | .01 | 6.24 | .10 | 57 | - |  |
| **Verbal Fluency** |  |  |  |  |  |  |  |  |  |
| Patients | 7 | .20 (.04; .36) | 2.40 | .02 | 8.31 | .22 | 34 |  | - |
| Controls ^b^ | - | - | - | - | - | - | - | - | - |

Abbreviations: k, number of studies. Q represents the Cochran’s Q value as a measure of between-study heterogeneity. *I^2^*  represents the percentage of variation between studies explained by heterogeneity (25% low heterogeneity, 50% moderate heterogeneity, 75% high heterogeneity). Bias represents the p-value of the egger’s test used to measure publication bias (reported when k=10 or more). The QM(p) is the result of the subgroup analysis comparing the change in cognition between patients and controls. *Denotes a significant difference between patients and controls (p<.05).

^b^Not enough studies assessing.
